# Evolutionary Diversification of Prey and Predator Species Facilitated by Asymmetric Interactions

**DOI:** 10.1371/journal.pone.0163753

**Published:** 2016-09-29

**Authors:** Jian Zu, Jinliang Wang, Gang Huang

**Affiliations:** 1 School of Mathematics and Statistics, Xi’an Jiaotong University, Xi’an, 710049, P.R. China; 2 Department of Ecology and Evolution, The University of Chicago, Chicago, IL 60637, United States of America; 3 School of Mathematical Science, Heilongjiang University, Harbin, 150080, P.R. China; 4 School of Mathematics and Physics, China University of Geosciences, Wuhan, 430074, P.R. China; Shanxi University, CHINA

## Abstract

We investigate the influence of asymmetric interactions on coevolutionary dynamics of a predator-prey system by using the theory of adaptive dynamics. We assume that the defense ability of prey and the attack ability of predators all can adaptively evolve, either caused by phenotypic plasticity or by behavioral choice, but there are certain costs in terms of their growth rate or death rate. The coevolutionary model is constructed from a deterministic approximation of random mutation-selection process. To sum up, if prey’s trade-off curve is globally weakly concave, then five outcomes of coevolution are demonstrated, which depend on the intensity and shape of asymmetric predator-prey interactions and predator’s trade-off shape. Firstly, we find that if there is a weakly decelerating cost and a weakly accelerating benefit for predator species, then evolutionary branching in the predator species may occur, but after branching further coevolution may lead to extinction of the predator species with a larger trait value. However, if there is a weakly accelerating cost and a weakly accelerating benefit for predator species, then evolutionary branching in the predator species is also possible and after branching the dimorphic predator can evolutionarily stably coexist with a monomorphic prey species. Secondly, if the asymmetric interactions become a little strong, then prey and predators will evolve to an evolutionarily stable equilibrium, at which they can stably coexist on a long-term timescale of evolution. Thirdly, if there is a weakly accelerating cost and a relatively strongly accelerating benefit for prey species, then evolutionary branching in the prey species is possible and the finally coevolutionary outcome contains a dimorphic prey and a monomorphic predator species. Fourthly, if the asymmetric interactions become more stronger, then predator-prey coevolution may lead to cycles in both traits and equilibrium population densities. The Red Queen dynamic is a possible outcome under asymmetric predator-prey interactions.

## Introduction

Predator-prey interactions are common in natural world and both prey and predator species have showed enormous diversity in their phenotypic traits, such as body size, weight, running velocity, or arms level [1–5]. However, the underlying evolutionary mechanisms and dynamical behaviors of prey and predator’s diversity are not well understood [1–16]. Previous studies revealed that the outcomes of predator-prey interactions often depend on their phenotypic traits which affect or indicate their interaction ability, such as running speed or arms level. Sometimes the predator-prey interactions can be strong enough for the predator to be a major component of the environment in which only the phenotypic trait of prey species is evolving and vice versa [1, 2, 7, 10, 17]. But evolutionary change in either species may evoke an evolutionary change in the other, which then changes the original trait value of the first species. Therefore, in general both prey and predators may experience adaptive changes in their phenotypic traits, either caused by phenotypic plasticity or by behavioral choice [3, 18].

The coevolution of phenotypic traits has motivated various kinds of theoretical models in a predator-prey community [1, 2, 7, 9, 10, 17–21]. Particularly, it is found that the predator-prey interactions are usually classified as symmetric or asymmetric [1, 2, 7, 10, 17, 21]. On the one hand, symmetric predator-prey interactions have the property that the interactions are the stronger the more similar prey and predator traits are, such as body size. The predator maximizes its rate of capture of the prey by matching the prey’s phenotype, and the prey has a “bidirectional” axis of vulnerability [3]. The larger predators gain more from large prey than they would from small prey, and small predators gain more from small prey. The predation efficiency of a predator is a symmetric function of prey and predator’s phenotypic traits. Selection pressure acts on predators and prey in the same direction. Under symmetric predator-prey interactions, it is found that evolutionary branching in prey species is possible and the branching in the prey can induce secondary branching in the predator species [1, 2, 10]. In addition, it is found that under certain conditions symmetric predator-prey interactions may lead to a Red Queen dynamic, in which the species may coexist with cyclic changes in their phenotypic traits [10, 17, 20, 21]. But in these studies, it is not found that evolutionary branching occurs firstly in the predator species and most of models assume that there is no intraspecific competition in the predator species.

On the other hand, asymmetric predator-prey interactions have the property that a higher predator ability increases the capture rate, while a higher prey ability reduces it. Here, the phenotypic traits are defensive or attack skills of two species. Both the vulnerability of prey and attack ability of predator are a “unidirectional” axis. In other words, the predator’s capture rate is an asymmetric function of the prey and predator’s phenotypic traits, which is an appropriate model for trait interactions such as speed-speed, weapon-armor, and toxin-antitoxin [3, 8, 18]. It is found that such asymmetric predator-prey interactions occur widely in nature, and is therefore likely to be an important driving force for species diversity [3, 8]. However, under asymmetric predator-prey interactions, the coevolutionary dynamics has little been discussed both empirically and theoretically. There have been few studies focusing on whether evolutionary branching in the prey and predator species will occur under asymmetric interactions and whether a Red Queen dynamic is a possible evolutionary outcome of such an evolutionary process.

In the present work, we will propose a coevolutionary model of phenotypic traits under asymmetric predator-prey interactions, and explore what coevolutionary outcomes are possible when a population of predators and a population of prey undergo asymmetric interactions. Specifically, three main questions will be addressed. First, starting from a monomorphic prey and predator species we will investigate under what conditions evolutionary branching in the prey and predator species will occur and after branching whether the dimorphic species can continue to coexist on a much longer timescale of evolution. Here, a monomorphic species means only one phenotypic trait exists in a population of a species. A dimorphic species means two different phenotypes occur in the same population of a species. Second, we will explore the ecological and evolutionary conditions that allow for a continuously stable strategy. Third, we will study whether coevolutionary cycles are possible under asymmetric predator-prey interactions. The last outcome corresponds to a Red Queen dynamic [7, 17]. In this study, we assume that mutations are rare and of small phenotypic effect. Our methods are mainly based on the theory of population dynamics and adaptive dynamics [19, 21–26]. Particularly, the stochastic mutation-selection process is approximately described by a canonical equation [10, 19, 22, 26]. However, we limit ourselves to phenotypic evolution under clonal reproduction.

This study is different from previous studies [10, 17, 21], with the following new features: (1) We assume that the predator’s attack ability and the defense ability of prey all evolve, either caused by phenotypic plasticity or by behavioral choice, but there are certain costs in terms of the growth rate of prey and the death rate of predators; (2) Both the prey and predators are assumed to subject to the effect of intraspecific competition; (3) The asymmetric capture rate function is modeled in a more flexible way, which may fit a wide range of asymmetric predator-prey interactions.

The rest of the paper is organized as follows. Next section, we will present the population dynamics of a predator-prey system and its coevolutionary dynamics. Afterwards, the five main coevolutionary outcomes will be presented. At the same time, numerical simulation examples are also presented to illustrate the feasibility of our main results. A brief discussion is given at the end of this paper.

## Materials and Methods

In this section, we first develop a population dynamics for the evolving predator and prey species. From this population model, the invasion fitness and coevolutionary model under asymmetric predator-prey interactions will be derived.

### Population dynamics

We assume that the initial resident community is composed of a monomorphic predator and a monomorphic prey species. The functional response of predator species is assumed to be linear, but there are intraspecific competitions in both prey and predators. Therefore, the population model is given by

$$\begin{array}{c} \left\{ \begin{array}{c} \frac{dN}{dt}=rN-kN^{2}-aNP,\\ \frac{dP}{dt}=baNP-mP-cP^{2}, \end{array}\right. \end{array}$$ (1)

where *N* and *P* are respectively the population densities of prey and predators at time *t*; *r* and *k* are respectively the per capita growth rate and intensity of intraspecific competition of prey in the absence of predation; *a* is the capture rate per unit time per unit prey density by an average predator; *b* is the conversion efficiency of ingested prey into new predators; *m* is the per capita death rate of predators; and *c* is the intensity of intraspecific competition among predators.

Moreover, we assume that both the defense ability of prey and the attack ability of predators can adaptively evolve. The defense ability of prey and attack ability of predators are respectively specified by a single phenotypic trait *x*_1_ and *x*_2_ of interest, such as running speed or arms level. For simplicity, by using of the transformation *log*(*l*/*l*_*min*_)/*log*(*l*_*max*_/*l*_*min*_), where *l* is the real trait value and *l*_*min*_ and *l*_*max*_ are respectively minimum and maximum trait values, we scale the phenotypic traits *x*_1_ and *x*_2_ such that *x*_1_ and *x*_2_ vary between 0 and 1 [27]. First, we assume that the capture rate *a* is an asymmetric function of prey trait *x*_1_ and predator trait *x*_2_, that is, *a* = *a*(*x*_1_ − *x*_2_) and *a*(*x*_1_ − *x*_2_) decreases as the difference between *x*_1_ and *x*_2_ increases, which is an applicable model for trait interactions such as speed-speed, weapon-armor, and toxin-antitoxin [3, 18, 28]. Specifically, if the trait value of prey *x*_1_ is much greater than that of predators *x*_2_, then *a*(*x*_1_ − *x*_2_) becomes very small and the prey can escape predation effectively. In contrast, if the trait value of predators *x*_2_ is much greater than that of prey *x*_1_, then the capture rate *a*(*x*_1_ − *x*_2_) becomes very large.

In order to maximize the population fitness, the prey and predators will change their phenotypic traits *x*_1_ and *x*_2_, but this may inevitably lead to a cost in terms of their other abilities. Therefore, we further assume that the growth rate of prey species *r* is a decreasing function of *x*_1_ (trade-off function), that is, *r*′(*x*_1_) < 0; and the death rate of predators *m* is an increasing function of *x*_2_ (trade-off function), that is, *m*′(*x*_2_) > 0. Under these assumptions, the population Model (1) is changed to

$$\begin{array}{c} \left\{ \begin{array}{c} \frac{dN}{dt}=r\left(x_{1}\right)N-kN^{2}-a\left(x_{1}-x_{2}\right)NP,\\ \frac{dP}{dt}=ba\left(x_{1}-x_{2}\right)NP-m\left(x_{2}\right)P-cP^{2}, \end{array}\right. \end{array}$$ (2)

where

$$\begin{array}{c} \frac{dr(x_{1})}{dx_{1}}<0,\;\frac{dm(x_{2})}{dx_{2}}>0\;and\;\frac{da(x_{1}-x_{2})}{d(x_{1}-x_{2})}<0,\;x_{1},\;x_{2}\in \left[0,1\right], \end{array}$$

and *r*(*x*_1_), *m*(*x*_2_) and *a*(*x*_1_ − *x*_2_) are respectively continuously differentiable functions with respect to *x*_1_, *x*_2_ and (*x*_1_ − *x*_2_). *a*(*x*_1_ − *x*_2_) and *r*(*x*_1_) might be a concave-convex decreasing function or a convex decreasing function or a more complex decreasing function; *m*(*x*_2_) might be a concave increasing function or a convex increasing function or a more complex increasing function, but the exact feature of these relationships may be unknown. However, all of the parameters are positive when *x*_1_, *x*_2_ ∈ [0, 1].

In addition, from the point view of biology, if a part of the trade-off curve *r*(*x*_1_) is convex, note that *r*(*x*_1_) is a decreasing function, then in this part each unit of improvement in the defense ability will come at an ever decreasing cost in terms of the growth rate of prey species. Therefore, in this case, we say there is a decelerating cost in the prey species. In contrast, if a part of the trade-off curve *r*(*x*_1_) is concave, then we say there is an accelerating cost in the prey species. Similarly, if a part of the trade-off curve *m*(*x*_2_) is convex, note that *m*(*x*_2_) is an increasing function, in this case their slopes are increasing, hence we say there is an accelerating cost in the predator species. However, if a part of the trade-off curve *m*(*x*_2_) is concave, then we say there is a decelerating cost in the predator species [29–31]. Moreover, if a part of the asymmetric capture rate curve *a*(*x*_1_ − *x*_2_) is convex, because *a*(*x*_1_ − *x*_2_) is a decreasing function, in this part each unit of improvement in the defense ability *x*_1_ will lead to a decreasing benefit in terms of the anti-predation efficiency. Therefore, in this case, we say there is a decelerating benefit for the prey species. But for the predator species, in this case each unit of improvement in the attack ability *x*_2_ will lead to an increasing benefit in terms of their predation efficiency. Hence we say there is an accelerating benefit for the predator species in this convex segment. On the contrary, if a part of the asymmetric capture rate curve *a*(*x*_1_ − *x*_2_) is concave, then we say there is an accelerating benefit for the prey species, but there is a decelerating benefit for the predator species in this concave part.

When

$$\begin{array}{c} br\left(x_{1}\right)a\left(x_{1}-x_{2}\right)>km\left(x_{2}\right), \end{array}$$ (3)

Model (2) admits a positive ecological equilibrium (N*(*x*_1_, *x*_2_), P*(*x*_1_, *x*_2_)), where

$$\begin{array}{c} \left\{ \begin{array}{c} N^{*}\left(x_{1},x_{2}\right)=\frac{m\left(x_{2}\right)a\left(x_{1}-x_{2}\right)+cr\left(x_{1}\right)}{ck+ba^{2}\left(x_{1}-x_{2}\right)},\\ P^{*}\left(x_{1},x_{2}\right)=\frac{br\left(x_{1}\right)a\left(x_{1}-x_{2}\right)-km\left(x_{2}\right)}{ck+ba^{2}\left(x_{1}-x_{2}\right)}, \end{array}\right. \end{array}$$ (4)

and it is globally asymptotically stable. On the ecological timescale, the prey and predators may not coexist for some trait values *x*_1_ and *x*_2_ and trade-off functions. Therefore, we define a feasible phenotype space **X** such that the Condition (3) holds and **X** is non-empty, so that the coevolution can occur. Based on Model (2), we next derive the invasion fitness for a mutant prey and a mutant predator species and propose a coevolutionary model.

### Coevolutionary dynamics

In order to study the coevolutionary dynamics of a predator-prey system, we make the following basic assumptions:

1. Mutations are rare and of small phenotypic effect.
2. Mutations occur infrequently such that a mutant strategy either has spread or has been excluded, and the population has reached its ecological equilibrium (N*(*x*_1_, *x*_2_), P*(*x*_1_, *x*_2_)) by the time the next mutant comes along, that is to say, we separate the ecological and evolutionary timescales.
3. There is either a mutant prey or a mutant predator, but not both at a time.

Under these assumptions, when a mutant prey with a slightly different trait *y*_1_ enters into the resident community at a low density, the invasion fitness [22] is given by

$$\begin{array}{c} f_{1}\left(y_{1},x_{1},x_{2}\right)=r\left(y_{1}\right)-kN^{*}\left(x_{1},x_{2}\right)-a\left(y_{1}-x_{2}\right)P^{*}\left(x_{1},x_{2}\right). \end{array}$$ (5)

The detailed method to derive the invasion fitness *f*_1_(*y*_1_, *x*_1_, *x*_2_) is described in S1 Appendix. We can see that if *f*_1_(*y*_1_, *x*_1_, *x*_2_) > 0, then the population density of mutant prey will initially increase, that is to say, the mutant prey can invade. Similarly, the invasion fitness for a mutant predator is given by

$$\begin{array}{c} f_{2}\left(y_{2},x_{1},x_{2}\right)=ba\left(x_{1}-y_{2}\right)N^{*}\left(x_{1},x_{2}\right)-m\left(y_{2}\right)-cP^{*}\left(x_{1},x_{2}\right). \end{array}$$ (6)

From Eqs (5) and (6), we can see that the resident traits and the equilibrium population densities of prey and predators affect their invasion fitness and will thus act as feedback variables in the coevolutionary process.

Furthermore, under above three assumptions, we can show that if *f*_1_(*y*_1_, *x*_1_, *x*_2_) > 0 and the trait *x*_1_ is not an evolutionarily singular strategy, then a successful invasion will cause a trait substitution of prey species (The detailed proof is moved to S2 Appendix). In other words, if there is no mutual invasibility and mutations are small and rare, a successful invader will replace the resident prey, and thus becomes a new resident prey [32–34]. Similarly, we can show that a successful invasion will cause a trait substitution of predator species.

Through continuing invasion and substitution, the prey and predators will keep on evolving. The direction of their coevolution is determined by selection gradients *g*_1_(*x*_1_, *x*_2_) and *g*_2_(*x*_1_, *x*_2_), which are given by

$$\begin{array}{c} \left\{ \begin{array}{c} g_{1}\left(x_{1},x_{2}\right)={\frac{\partial f_{1}\left(y_{1},x_{1},x_{2}\right)}{\partial y_{1}}|}_{y_{1}=x_{1}}=r^{\prime }\left(x_{1}\right)-a^{\prime }\left(x_{1}-x_{2}\right)P^{*}\left(x_{1},x_{2}\right),\\ g_{2}\left(x_{1},x_{2}\right)={\frac{\partial f_{2}\left(y_{2},x_{1},x_{2}\right)}{\partial y_{2}}|}_{y_{2}=x_{2}}=-ba^{\prime }\left(x_{1}-x_{2}\right)N^{*}\left(x_{1},x_{2}\right)-m^{\prime }\left(x_{2}\right), \end{array}\right. \end{array}$$ (7)

where

$$\begin{array}{c} r^{\prime }\left(x_{1}\right)={\frac{dr(y_{1})}{dy_{1}}|}_{y_{1}=x_{1}},\;m^{\prime }\left(x_{2}\right)={\frac{dm(y_{2})}{dy_{2}}|}_{y_{2}=x_{2}},\;a^{\prime }\left(x_{1}-x_{2}\right)={\frac{\partial a(y_{1}-x_{2})}{\partial y_{1}}|}_{y_{1}=x_{1}}. \end{array}$$ (8)

Particularly, if the mutation processes are homogeneous and mutations are rare and small, by the results of Dieckmann and Law [26], the step by step evolution can be approximately described by the following canonical equation

$$\begin{array}{c} \left\{ \begin{array}{c} \frac{dx_{1}}{d\tau }=m_{1}\left(x_{1},x_{2}\right)g_{1}\left(x_{1},x_{2}\right),\\ \frac{dx_{2}}{d\tau }=m_{2}\left(x_{1},x_{2}\right)g_{2}\left(x_{1},x_{2}\right), \end{array}\right. \end{array}$$ (9)

where time *τ* spans the evolutionary timescale, *g*_1_(*x*_1_, *x*_2_) and *g*_2_(*x*_1_, *x*_2_) are selection gradients described as in Eq (8), *m*_1_(*x*_1_, *x*_2_) and *m*_2_(*x*_1_, *x*_2_) are respectively the evolutionary rates of prey and predator species, where

$$\begin{array}{c} \left\{ \begin{array}{c} m_{1}\left(x_{1},x_{2}\right)=\frac{1}{2}\mu_{1}\sigma_{1}^{2}N^{*}\left(x_{1},x_{2}\right),\\ m_{2}\left(x_{1},x_{2}\right)=\frac{1}{2}\mu_{2}\sigma_{2}^{2}P^{*}\left(x_{1},x_{2}\right), \end{array}\right. \end{array}$$ (10)

*μ*_1_ (*μ*_2_) is the probability that a birth event in the prey (predator) species is a mutant, $\sigma_{1}^{2}$ and $\sigma_{2}^{2}$ are respectively the variances of phenotypic effect of prey and predator mutations. N*(*x*_1_, *x*_2_) and P*(*x*_1_, *x*_2_) are the ecological equilibrium population densities of prey and predator species. Model (9) tells us how the expected values of traits *x*_1_ and *x*_2_ will change. We next investigate the eventually outcomes of such a coevolutionary process.

## Results

### Continuously stable strategy

If there exists a pair of traits $\left(x_{1}^{*},x_{2}^{*}\right)$ such that

$$\begin{array}{c} \left\{ \begin{array}{c} g_{1}\left(x_{1}^{*},x_{2}^{*}\right)=r^{\prime }\left(x_{1}^{*}\right)-a^{\prime }\left(x_{1}^{*}-x_{2}^{*}\right)P^{*}\left(x_{1}^{*},x_{2}^{*}\right)=0,\\ g_{2}\left(x_{1}^{*},x_{2}^{*}\right)=-ba^{\prime }\left(x_{1}^{*}-x_{2}^{*}\right)N^{*}\left(x_{1}^{*},x_{2}^{*}\right)-m^{\prime }\left(x_{2}^{*}\right)=0, \end{array}\right. \end{array}$$ (11)

then $\left(x_{1}^{*},x_{2}^{*}\right)$ is called an ‘evolutionarily singular coalition’ [19, 23], at which the coevolution may come to a halt. Notice that *r*′(*x*_1_) < 0 and *m*′(*x*_2_) > 0, from Eq (11), it can be seen that $a^{\prime }\left(x_{1}^{*}-x_{2}^{*}\right)$ must be negative otherwise the selection gradients cannot be zero at $\left(x_{1}^{*},x_{2}^{*}\right)$. In general, the predator-prey coevolution will come to a halt at the singular coalition $\left(x_{1}^{*},x_{2}^{*}\right)$ if the following two stability criteria are stisfied [19, 35–37].

First, the singular coalition is convergence stable such that the directional coevolution can approach it. Whether the singular coalition $\left(x_{1}^{*},x_{2}^{*}\right)$ is convergence stable or not can be seen from the Jacobian matrix of evolutionary dynamics Eq (9) at this point [7, 10, 38]. The Jacobian matrix *J*_1_ of evolutionary dynamics Eq (9) evaluated at $\left(x_{1}^{*},x_{2}^{*}\right)$ is given by

J1=[m1(x1,x2)∂g1(x1,x2)∂x1m1(x1,x2)∂g1(x1,x2)∂x2m2(x1,x2)∂g2(x1,x2)∂x1m2(x1,x2)∂g2(x1,x2)∂x2]x1=x1*,x2=x2*.

If the determinant of the Jacobian matrix is positive (*det*(*J*_1_) > 0) and its trace is negative (*tr*(*J*_1_) < 0), then the singular coalition $\left(x_{1}^{*},x_{2}^{*}\right)$ is locally convergence stable.

Second, the singular coalition is evolutionarily stable so that $\left(x_{1}^{*},x_{2}^{*}\right)$ can not be invaded by any nearby strategy. The evolutionary stability can be estimated by calculating the second derivative of the invasion fitness with respect to mutant trait value and evaluating at the singular coalition $\left(x_{1}^{*},x_{2}^{*}\right)$ [19, 35, 39]. Specifically, if

{∂2f1(y1,x1,x2)∂y12|x2=x2*,y1=x1=x1*=r′′(x1*)-a′′(x1*-x2*)P*(x1*,x2*)<0,∂2f2(y2,x1,x2)∂y22|x1=x1*,y2=x2=x2*=ba′′(x1*-x2*)N*(x1*,x2*)-m′′(x2*)<0, (12)

where

r′′(x1*)=d2r(y1)dy12|y1=x1*,m′′(x2*)=d2m(y2)dy22|y2=x2*,a′′(x1*-x2*)=∂2a(y1-x2)∂y12|y1=x1*,x2=x2*,,

then the singular coalition $\left(x_{1}^{*},x_{2}^{*}\right)$ is locally evolutionarily stable.

If the singular coalition $\left(x_{1}^{*},x_{2}^{*}\right)$ is both convergence stable and evolutionarily stable, it is called a continuously stable strategy (CSS) [35, 39], once it is established the predator-prey system cannot be invaded by any nearby strategy. Given specific functions *r*(*x*_1_), *m*(*x*_2_) and *a*(*x*_1_ − *x*_2_), the Jacobian matrix *J*_1_ and Condition (12) can be easily analyzed numerically. Therefore, combining the conditions of convergence stability and evolutionary stability, we obtain the following result.

**Proposition 1** *Assume that* Condition (3) *holds. For the evolutionarily singular coalition* $\left(x_{1}^{*},x_{2}^{*}\right)$ *of* Model (9), *if* *det*(*J*_1_) > 0, *tr*(*J*_1_) < 0 *and* Condition (12) *is satisfied, then the singular coalition* $\left(x_{1}^{*},x_{2}^{*}\right)$ *is a continuously stable strategy*.

From the Jacobian matrix *J*_1_ and Condition (12), we can see that whether the singular coalition $\left(x_{1}^{*},x_{2}^{*}\right)$ is continuously stable depends on the curvatures of trade-off functions at this singular coalition and the relative intensity of asymmetric interactions. Moreover, it can be seen that the equilibrium population densities of prey and predator species also play an important role in determining whether the singular coalition $\left(x_{1}^{*},x_{2}^{*}\right)$ is continuously stable. At the continuously stable strategy $\left(x_{1}^{*},x_{2}^{*}\right)$, both prey and predators can stably coexist on a long-term timescale of evolution. Therefore, an evolutionarily singular coalition that is continuously stable represents an eventual outcome of such a coevolutionary process. In this case, the finally evolutionary outcome contains a monomorphic prey and a monomorphic predator species.

### Evolutionary branching

However, evolutionary branching in the prey or predator species may occur when one of the opposite inequalities of Eq (12) holds. Generally speaking, in two-dimensional evolutionary dynamics, an ‘evolutionarily singular coalition’ that is convergence stable but for which at least one strategy lacks evolutionary stability and allows for mutual invasibility nearby will lead to evolutionary branching [9, 10, 17, 19, 23]. Therefore, we obtain the following results on the evolutionary branching of prey and predator species.

Firstly, if the singular coalition $\left(x_{1}^{*},x_{2}^{*}\right)$ is convergence stable and the predator singular strategy $x_{2}^{*}$ is evolutionarily stable, but the prey singular strategy $x_{1}^{*}$ is not evolutionarily stable and allows for mutual invasibility of the mutant prey and resident prey, i.e.,

∂2f1(y1,x1,x2)∂y12|x2=x2*,y1=x1=x1*=r′′(x1*)-a′′(x1*-x2*)P*(x1*,x2*)>0, (13)

and

∂2f1(y1,x1,x2)∂x12|x2=x2*,y1=x1=x1*>-∂2f1(y1,x1,x2)∂y12|x2=x2*,y1=x1=x1*, (14)

then evolutionary branching in the prey species will occur.

Similarly, we can see that if the singular coalition $\left(x_{1}^{*},x_{2}^{*}\right)$ is convergence stable and the singular strategy $x_{1}^{*}$ of prey is evolutionarily stable, but the predator strategy $x_{2}^{*}$ is not evolutionarily stable and allows for mutual invasibility, i.e.,

∂2f2(y2,x1,x2)∂y22|x1=x1*,y2=x2=x2*=ba′′(x1*-x2*)N*(x1*,x2*)-m′′(x2*)>0, (15)

and

∂2f2(y2,x1,x2)∂x22|x1=x1*,y2=x2=x2*>-∂2f2(y2,x1,x2)∂y22|x1=x1*,y2=x2=x2*, (16)

then evolutionary branching in the predator species will occur. The above conditions are also easily estimated numerically once the specific functions *r*(*x*_1_), *m*(*x*_2_) and *a*(*x*_1_ − *x*_2_) are given. Therefore, we obtain the following results on the evolutionary branching of prey and predator species.

**Proposition 2** *Assume that* Condition (3) *holds. For the evolutionarily singular coalition* $\left(x_{1}^{*},x_{2}^{*}\right)$ *of* Model (9),

1. *if* *det*(*J*_1_) > 0, *tr*(*J*_1_) < 0 *and conditions* (13), (14) *and the second condition of* Eq (12) *are satisfied, then evolutionary branching in the prey species will occur*;
2. *if* *det*(*J*_1_) > 0, *tr*(*J*_1_) < 0 *and conditions* (15), (16) *and the first condition of* Eq (12) *are satisfied, then evolutionary branching in the predator species will occur*.

From the above analysis, it can be seen that whether evolutionary branching will occur in the prey or predator species depends on the shape and relative intensity of asymmetric interactions and the trade-off shapes. In addition, from conditions (13) and (15), we can see that the ecological equilibrium population densities of prey and predator species are also crucial in determining whether the evolutionary branching occurs. Particularly, from Condition (13), it can be seen that if the growth rate of prey *r*(*x*_1_) is weakly concave at $x_{1}^{*}$, but the capture rate *a*(*x*_1_ − *x*_2_) is relatively strongly concave at $\left(x_{1}^{*}-x_{2}^{*}\right)$, then evolutionary branching in the prey species might be possible. In other words, if there is a weakly accelerating cost in the prey species, but at the same time there is a strongly accelerating benefit in terms of anti-predation efficiency, then evolutionary branching in the prey species might occur. Besides, from Condition (15), we can see that if the capture rate *a*(*x*_1_ − *x*_2_) is convex at $\left(x_{1}^{*}-x_{2}^{*}\right)$ and the death rate of predators *m*(*x*_2_) is concave at $x_{2}^{*}$, then evolutionary branching in the predator species might be possible. That is to say, if there is an accelerating benefit and a decelerating cost in the predator species, then evolutionary branching in the predator species might occur. However, if *k* = 0 or *c* = 0, that is to say, there is no intraspecific competition in the prey or predator species, then evolutionary branching cannot occur in the prey or predator species. Because in this case, two different types of prey (predators) cannot stably coexist with a single predator (prey) species when their functional responses are linear.

It should be noted that if both singular strategies lack evolutionary stability and they evolve with different speed, then the faster of them can undergo branching. As branching in one strategy generally changes the frequency-dependent fitness function, the slower evolving strategy may no longer be near a branching point, i.e., they may have missed the opportunity for branching [23].

If evolutionary branching is possible in the prey and predator species, then the prey and predators will firstly evolve towards the singular coalition $\left(x_{1}^{*},x_{2}^{*}\right)$ and near $\left(x_{1}^{*},x_{2}^{*}\right)$, the prey or predators will split up into two diverging types. After branching has occurred in the prey or predator species, it becomes more interesting to investigate the further coevolutionary dynamics of a one-predator-two-prey system or a two-predator-one-prey system and find the eventual outcome of such an evolutionary process.

### Evolutionarily stable coexistence of one predator and two prey species

In case of the prey species firstly branches into two different phenotypes, *N*_1_ and *N*_2_, one with strategy *x*_11_ and the other with a slightly different strategy *x*_12_, the population dynamics is given by

$$\begin{array}{c} \left\{ \begin{array}{c} \frac{dN_{1}}{dt}=r\left(x_{11}\right)N_{1}-k\left(N_{1}+N_{2}\right)N_{1}-a\left(x_{11}-x_{2}\right)N_{1}P,\\ \frac{dN_{2}}{dt}=r\left(x_{12}\right)N_{2}-k\left(N_{1}+N_{2}\right)N_{2}-a\left(x_{12}-x_{2}\right)N_{2}P,\\ \frac{dP}{dt}=ba\left(x_{11}-x_{2}\right)N_{1}P+ba\left(x_{12}-x_{2}\right)N_{2}P-m\left(x_{2}\right)P-cP^{2}, \end{array}\right. \end{array}$$ (17)

where *N*_*i*_ (*i* = 1, 2) denote population densities of prey 1 and prey 2 at time *t*.

Set

$$\begin{array}{ccc} b_{1} & = & km\left(x_{2}\right)\left(a\left(x_{11}-x_{2}\right)-a\left(x_{12}-x_{2}\right)\right)+ck\left(r\left(x_{11}\right)-r\left(x_{12}\right)\right)\\ & & +ba\left(x_{12}-x_{2}\right)\left(r\left(x_{11}\right)a\left(x_{12}-x_{2}\right)-r\left(x_{12}\right)a\left(x_{11}-x_{2}\right)\right),\\ b_{2} & = & km\left(x_{2}\right)\left(a\left(x_{12}-x_{2}\right)-a\left(x_{11}-x_{2}\right)\right)+ck\left(r\left(x_{12}\right)-r\left(x_{11}\right)\right)\\ & & +ba\left(x_{11}-x_{2}\right)\left(r\left(x_{12}\right)a\left(x_{11}-x_{2}\right)-r\left(x_{11}\right)a\left(x_{12}-x_{2}\right)\right),\\ b_{3} & = & \left(r\left(x_{11}\right)-r\left(x_{12}\right)\right)\left(a\left(x_{11}-x_{2}\right)-a\left(x_{12}-x_{2}\right)\right), \end{array}$$ (18)

when *b*_*i*_ > 0 (*i* = 1, 2, 3), we obtain a positive ecological equilibrium $\left(N_{1}^{*}\left(x\right),N_{2}^{*}\left(x\right),P^{*}\left(x\right)\right)$, which is globally asymptotically stable (The mathematically rigorous proof is explained in S3 Appendix), where **x** = (*x*_11_, *x*_12_, *x*_2_) and

$$\begin{array}{c} \left\{ \begin{array}{c} N_{1}^{*}\left(x\right)=\frac{b_{1}}{bk{\left(a\left(x_{11}-x_{2}\right)-a\left(x_{12}-x_{2}\right)\right)}^{2}},\\ N_{2}^{*}\left(x\right)=\frac{b_{2}}{bk{\left(a\left(x_{11}-x_{2}\right)-a\left(x_{12}-x_{2}\right)\right)}^{2}},\\ P^{*}\left(x\right)=\frac{r\left(x_{11}\right)-r\left(x_{12}\right)}{a\left(x_{11}-x_{2}\right)-a\left(x_{12}-x_{2}\right)}. \end{array}\right. \end{array}$$ (19)

We assume that there is either a mutant predator or a mutant prey, and the mutant prey either arises from prey 1 or from prey 2, but not both at a time. Therefore, by the same derivation as in the ‘Materials and Methods’ section, when a mutant prey with a slightly different trait *y*_1_ appears in the resident system at a low density, the invasion fitness is given by

$$\begin{array}{c} h_{1}\left(y_{1},x\right)=r\left(y_{1}\right)-k\left(N_{1}^{*}\left(x\right)+N_{2}^{*}\left(x\right)\right)-a\left(y_{1}-x_{2}\right)P^{*}\left(x\right). \end{array}$$ (20)

Likewise, the invasion fitness for a mutant predator is

$$\begin{array}{ccc} h_{2}\left(y_{2},x\right) & = & ba\left(x_{11}-y_{2}\right)N_{1}^{*}\left(x\right)+ba\left(x_{12}-y_{2}\right)N_{2}^{*}\left(x\right)-m\left(y_{2}\right)-cP^{*}\left(x\right). \end{array}$$ (21)

Therefore, we can calculate the selection gradients as follows

$$\begin{array}{c} \left\{ \begin{array}{c} g_{11}\left(x\right)={\frac{\partial h_{1}\left(y_{1},x\right)}{\partial y_{1}}|}_{y_{1}=x_{11}}=r^{\prime }\left(x_{11}\right)-a^{\prime }\left(x_{11}-x_{2}\right)P^{*}\left(x\right),\\ g_{12}\left(x\right)={\frac{\partial h_{1}\left(y_{1},x\right)}{\partial y_{1}}|}_{y_{1}=x_{12}}=r^{\prime }\left(x_{12}\right)-a^{\prime }\left(x_{12}-x_{2}\right)P^{*}\left(x\right),\\ g_{2}\left(x\right)={\frac{\partial h_{2}\left(y_{2},x\right)}{\partial y_{2}}|}_{y_{2}=x_{2}}=-ba^{\prime }\left(x_{11}-x_{2}\right)N_{1}^{*}\left(x\right)-ba^{\prime }\left(x_{12}-x_{2}\right)N_{2}^{*}\left(x\right)-m^{\prime }\left(x_{2}\right). \end{array}\right. \end{array}$$ (22)

If the mutation processes are homogeneous and mutations are rare and sufficiently small, the coevolutionary dynamics of traits *x*_11_, *x*_12_ and *x*_2_ is given by

$$\begin{array}{c} \left\{ \begin{array}{c} \frac{dx_{11}}{d\tau }=\frac{1}{2}\mu_{11}\sigma_{11}^{2}N_{1}^{*}\left(x\right)g_{11}\left(x\right),\\ \frac{dx_{12}}{d\tau }=\frac{1}{2}\mu_{12}\sigma_{12}^{2}N_{2}^{*}\left(x\right)g_{12}\left(x\right),\\ \frac{dx_{2}}{d\tau }=\frac{1}{2}\mu_{2}\sigma_{2}^{2}P^{*}\left(x\right)g_{2}\left(x\right), \end{array}\right. \end{array}$$ (23)

where *g*_1*i*_(**x**)(*i* = 1, 2) and *g*_2_(**x**) are selection gradients described as in Eq (22) [26]. *μ*_1*i*_, (*i* = 1, 2) and *μ*_2_ are the probabilities that a birth event in the prey 1 or prey 2, or predator species is a mutant; $\sigma_{1i}^{2},\left(i=1,2\right)$ and $\sigma_{2}^{2}$ are respectively the variances of the phenotypic effect of prey 1, prey 2 and predator mutations. $N_{i}^{*}\left(x\right),\left(i=1,2\right)$ and P*(**x**) are the ecological equilibrium population densities of the prey 1, prey 2 and predators. Model (23) tells us how the expected values of traits *x*_11_, *x*_12_ and *x*_2_ will change.

Setting the right-hand sides of Eq (22) to 0, we can obtain an evolutionarily singular strategy $x^{*}=\left(x_{11}^{*},x_{12}^{*},x_{2}^{*}\right)$ of Model (23). The convergence stability of this singular strategy can be studied by numerical analysis and computer simulations. Its evolutionary stability is determined by the following conditions:

{∂2h1(y1,x)∂y12|y1=x1i=x1i*x2=x2*,x1j=x1j*=r′′(x1i*)-a′′(x1i*-x2*)P*(x*)<0,(i,j=1,2,i≠j),∂2h2(y2,x)∂y22|y2=x2=x2*x11=x11*,x12=x12*=ba′′(x11*-x2*)N1*(x*)+ba′′(x12*-x2*)N2*(x*)-m′′(x2*)<0. (24)

To sum up, we obtain the following result.

**Proposition 3** *Assume that* *b*_*i*_ (*i* = 1, 2, 3) *in* Eq (18) *are positive. For the evolutionarily singular strategy* $\left(x_{11}^{*},x_{12}^{*},x_{2}^{*}\right)$ *of* Model (23), *if it is convergence stable and* Condition (24) *is satisfied, then it is a continuously stable strategy*.

From Eqs (23) and (24), we can see that whether the singular strategy $\left(x_{11}^{*},x_{12}^{*},x_{2}^{*}\right)$ is continuously stable depends on the intensity and shape of asymmetric interactions and the curvatures of trade-off functions, which also depends on the equilibrium population densities of prey 1, prey 2 and predator species. Specially, combining with the analysis of evolutionary branching in the prey species, we can see that if there is a weakly accelerating cost in the prey species, and meanwhile there is a relatively strongly accelerating benefit in terms of anti-predation efficiency, then evolutionary branching might occur in the prey species. After branching the two prey and one predator species may converge to an evolutionarily stable equilibrium such that Condition (24) is satisfied. If the singular strategy $\left(x_{11}^{*},x_{12}^{*},x_{2}^{*}\right)$ is both convergence stable and evolutionarily stable, it represents an eventual outcome of such a coevolutionary process, at which the two prey and one predator species can stably coexist on a much longer evolutionary timescale. Therefore, in this case, the finally evolutionary outcome contains a dimorphic prey and a monomorphic predator species.

However, if the singular strategy $\left(x_{11}^{*},x_{12}^{*},x_{2}^{*}\right)$ is convergence stable, but one of the two prey strategies lacks evolutionary stability and allows for mutual invasibility, then a further evolutionary branching in the prey species might occur. In this case, we can use the same methods as described above to study the further coevolution of strategies in the polymorphic prey population, but a full exploration of which is beyond the scope of this paper. In contrast, if the predator strategy firstly lacks evolutionary stability and allows for mutual invasibility, then we will use the method described in the next subsection to discuss the coevolutionary dynamics of a dimorphic predator species and find the eventual outcome of such a coevolutionary process.

### Evolutionarily stable coexistence of one prey and two predator species

If the predator species firstly splits into two different phenotypes, *P*_1_ and *P*_2_, the population dynamics becomes

$$\begin{array}{c} \left\{ \begin{array}{c} \frac{dN}{dt}=r\left(x_{1}\right)N-kN^{2}-a\left(x_{1}-x_{21}\right)NP_{1}-a\left(x_{1}-x_{22}\right)NP_{2},\\ \frac{dP_{1}}{dt}=ba\left(x_{1}-x_{21}\right)NP_{1}-m\left(x_{21}\right)P_{1}-c\left(P_{1}+P_{2}\right)P_{1},\\ \frac{dP_{2}}{dt}=ba\left(x_{1}-x_{22}\right)NP_{2}-m\left(x_{22}\right)P_{2}-c\left(P_{1}+P_{2}\right)P_{2}, \end{array}\right. \end{array}$$ (25)

where *P*_*i*_ (*i* = 1, 2) denote population densities of predator 1 and predator 2 at time *t*, *x*_21_ and *x*_22_ denote respectively the strategies of predator 1 and predator 2.

Set

$$\begin{array}{ccc} c_{1} & = & bcr\left(x_{1}\right)\left(a\left(x_{1}-x_{21}\right)-a\left(x_{1}-x_{22}\right)\right)+ck\left(m\left(x_{22}\right)-m\left(x_{21}\right)\right)\\ & & +ba\left(x_{1}-x_{22}\right)\left(m\left(x_{22}\right)a\left(x_{1}-x_{21}\right)-m\left(x_{21}\right)a\left(x_{1}-x_{22}\right)\right),\\ c_{2} & = & bcr\left(x_{1}\right)\left(a\left(x_{1}-x_{22}\right)-a\left(x_{1}-x_{21}\right)\right)+ck\left(m\left(x_{21}\right)-m\left(x_{22}\right)\right)\\ & & +ba\left(x_{1}-x_{21}\right)\left(m\left(x_{21}\right)a\left(x_{1}-x_{22}\right)-m\left(x_{22}\right)a\left(x_{1}-x_{21}\right)\right),\\ c_{3} & = & \left(m\left(x_{21}\right)-m\left(x_{22}\right)\right)\left(a\left(x_{1}-x_{21}\right)-a\left(x_{1}-x_{22}\right)\right), \end{array}$$ (26)

when *c*_*i*_ > 0 (*i* = 1, 2, 3), there exists a positive ecological equilibrium $\left(N^{*}\left(x\right),P_{1}^{*}\left(x\right),P_{2}^{*}\left(x\right)\right)$, which is globally asymptotically stable (The detailed proof is explained in S4 Appendix), where **x** = (*x*_1_, *x*_21_, *x*_22_) and

$$\begin{array}{c} \left\{ \begin{array}{c} N^{*}\left(x\right)=\frac{m\left(x_{21}\right)-m\left(x_{22}\right)}{b(a\left(x_{1}-x_{21}\right)-a\left(x_{1}-x_{22}\right))},\\ P_{1}^{*}\left(x\right)=\frac{c_{1}}{bc{\left(a\left(x_{1}-x_{21}\right)-a\left(x_{1}-x_{22}\right)\right)}^{2}},\\ P_{2}^{*}\left(x\right)=\frac{c_{2}}{bc{\left(a\left(x_{1}-x_{21}\right)-a\left(x_{1}-x_{22}\right)\right)}^{2}}. \end{array}\right. \end{array}$$ (27)

By using the same methods as in ‘Materials and Methods’ section, we obtain the invasion fitness for a mutant prey and a mutant predator

$$\begin{array}{ccc} q_{1}\left(y_{1},x\right) & = & r\left(y_{1}\right)-kN^{*}-a\left(y_{1}-x_{21}\right)P_{1}^{*}\left(x\right)-a\left(y_{1}-x_{22}\right)P_{2}^{*}\left(x\right),\\ q_{2}\left(y_{2},x\right) & = & ba\left(x_{1}-y_{2}\right)N^{*}\left(x\right)-m\left(y_{2}\right)-c\left(P_{1}^{*}\left(x\right)+P_{2}^{*}\left(x\right)\right), \end{array}$$ (28)

where *y*_1_ and *y*_2_ denote respectively the strategies of a mutant prey and a mutant predator.

By the results of Dieckmann and Law [26], the coevolutionary dynamics of traits *x*_1_, *x*_21_ and *x*_22_ is given by

$$\begin{array}{c} \left\{ \begin{array}{c} \frac{dx_{1}}{d\tau }=\frac{1}{2}\mu_{1}\sigma_{1}^{2}N^{*}\left(x\right)g_{1}\left(x\right),\\ \frac{dx_{21}}{d\tau }=\frac{1}{2}\mu_{21}\sigma_{21}^{2}P_{1}^{*}\left(x\right)g_{21}\left(x\right),\\ \frac{dx_{22}}{d\tau }=\frac{1}{2}\mu_{22}\sigma_{22}^{2}P_{2}^{*}\left(x\right)g_{22}\left(x\right), \end{array}\right. \end{array}$$ (29)

where

$$\begin{array}{c} \left\{ \begin{array}{c} g_{1}\left(x\right)={\frac{\partial q_{1}\left(y_{1},x\right)}{\partial y_{1}}|}_{y_{1}=x_{1}}=r^{\prime }\left(x_{1}\right)-a^{\prime }\left(x_{1}-x_{21}\right)P_{1}^{*}\left(x\right)-a^{\prime }\left(x_{1}-x_{22}\right)P_{2}^{*}\left(x\right),\\ g_{21}\left(x\right)={\frac{\partial q_{2}\left(y_{2},x\right)}{\partial y_{2}}|}_{y_{2}=x_{21}}=-ba^{\prime }\left(x_{1}-x_{21}\right)N^{*}\left(x\right)-m^{\prime }\left(x_{21}\right),\\ g_{22}\left(x\right)={\frac{\partial q_{2}\left(y_{2},x\right)}{\partial y_{2}}|}_{y_{2}=x_{22}}=-ba^{\prime }\left(x_{1}-x_{22}\right)N^{*}\left(x\right)-m^{\prime }\left(x_{22}\right), \end{array}\right. \end{array}$$ (30)

*μ*_1_ and *μ*_2*i*_, (*i* = 1, 2) denote the probabilities that a birth event in the prey or predator 1, or predator 2 species is a mutant; $\sigma_{1}^{2}$ and $\sigma_{2i}^{2},\left(i=1,2\right)$ denote respectively the variances of phenotypic effect of prey, predator 1 and predator 2 mutations. N*(**x**) and $P_{i}^{*}\left(x\right),\left(i=1,2\right)$ denote the ecological equilibrium population densities of prey, predator 1 and predator 2. Model (29) tells us how the expected values of traits *x*_1_, *x*_21_ and *x*_22_ will change.

Setting the right-hand sides of Eq (30) to 0, we can obtain an evolutionarily singular strategy $x^{*}=\left(x_{1}^{*},x_{21}^{*},x_{22}^{*}\right)$ of Model (29). The convergence stability of this singular strategy can be calculated numerically. The evolutionary stability of this singular strategy is determined by the following conditions:

{∂2q1(y1,x)∂y12|y1=x1=x1*x21=x21*,x22=x22*=r′′(x1*)-a′′(x1*-x21*)P1*(x*)-a′′(x1*-x22*)P2*(x*)<0,∂2q2(y2,x)∂y22|y2=x2i=x2i*x1=x1*,x2j=x2j*=ba′′(x1*-x2i*)N*(x*)-m′′(x2i*)<0,(i,j=1,2,i≠j). (31)

In summary, we obtain the following result.

**Proposition 4** *Assume that* *c*_*i*_ (*i* = 1, 2, 3) *in* Eq (26) *are positive. For the evolutionarily singular strategy* $\left(x_{1}^{*},x_{21}^{*},x_{22}^{*}\right)$ *of* Model (29), *if it is convergence stable and* Condition (31) *is satisfied, then it is a continuously stable strategy*.

From Eqs (30) and (31), we can see that whether the singular strategy x* is continuously stable depends not only on the strengths and shapes of asymmetric interactions and trade-off shapes, but also on the equilibrium population densities of predator 1, predator 2 and prey species at this singular strategy x*. In particular, combining with the evolutionary branching analysis, we can see that if the predator’s trade-off curve *m*(*x*_2_) is globally concave and there is an accelerating benefit for the predator species, then evolutionary branching in the predator species might occur. But after branching further coevolution may lead to extinction of the predator species with a larger trait value, which corresponds to an evolutionary murder [25, 34, 40]. This might be due to the relatively higher cost of a larger trait value. However, if the predator’s trade-off curve *m*(*x*_2_) is globally convex, that is, the cost of a larger trait is relatively lower, but meanwhile there is an accelerating benefit for the predator species, then evolutionary branching in the predator species might also occur. In this case after branching the two predator and one prey species may converge to an evolutionarily stable equilibrium such that Condition (31) is satisfied. If the singular strategy $\left(x_{1}^{*},x_{21}^{*},x_{22}^{*}\right)$ is both convergence stable and evolutionarily stable, it represents an eventual outcome of such a coevolutionary process. The finally evolutionary outcome may contain a dimorphic predator and a monomorphic prey species. In addition, if the further evolutionary branching in the predator species occurs, we can use the same method as above to explore the further coevolution of a polymorphic predator population and find the eventual outcome of such a coevolutionary process. Repeated evolutionary branching may lead to high levels of polymorphism in the predator species, but a full exploration of which is beyond the scope of this paper.

### Evolutionary cycling

More interestingly, from the Jacobian matrix *J*_1_, we can see that if the asymmetric predator-prey interactions become relatively stronger, then the evolutionarily singular coalition $\left(x_{1}^{*},x_{2}^{*}\right)$ may become unstable, in this case Model (9) may admits a Hopf bifurcation, that is, the prey and predators will evolve to a stable limit cycle, the evolutionary cycling is a possible outcome of such a coevolutionary process. This phenomenon corresponds to a Red Queen dynamics, in which the relatively stronger asymmetric interactions between prey and predators can keep their phenotypic traits evolving infinitely [17]. Because of the complex nonlinearity, this result can only be analyzed numerically. Therefore, in the next section, we give some numerical simulation examples to illustrate the feasibility of our main results.

## Examples

In this section, through numerical analysis and computer simulation, we show that depending on the intensity and shape of asymmetric predator-prey interactions and the shape of predator’s trade-off curve, the evolutionary branching in the prey and predator species and evolutionary cycling are possible outcomes under asymmetric interactions.

**Fig 1:**
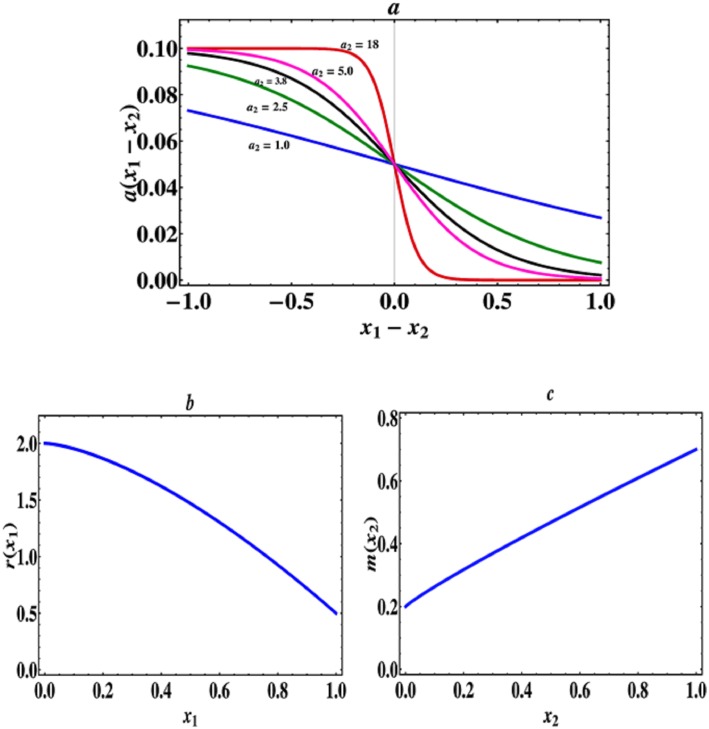
The concave-convex capture rate function and two trade-off functions. (a) Asymmetric capture rate *a*(*x*_1_ − *x*_2_) as a function of phenotypic difference (*x*_1_ − *x*_2_), where *a*_0_ = 0.1, *a*_1_ = 1.0. (b) The growth rate of prey *r*(*x*_1_) (trade-off function), where *r*_0_ = 0.5, *r*_1_ = 1.5, *r*_2_ = 1.5. (c) The death rate of predators *m*(*x*_2_) (trade-off function), where *m*_0_ = 0.2, *m*_1_ = 0.5, *m*_2_ = 0.9.

As an example, we take the following asymmetric capture rate function [23]

$$\begin{array}{c} a\left(x_{1}-x_{2}\right)=\frac{a_{0}}{1+a_{1}\exp\left(a_{2}\left(x_{1}-x_{2}\right)\right)}, \end{array}$$ (32)

which is a monotonically decreasing function with respect to (*x*_1_ − *x*_2_), where *a*_0_ is the maximum strength of predator-prey interactions and *a*_2_ measures the intensity of asymmetric interactions, the larger *a*_2_ is, the stronger the asymmetric interactions will be (see Fig 1a). This function can fit a variety of asymmetric interactions. Particularly, if *a*_1_ = 1.0, then there is an inflection point at *x*_1_ − *x*_2_ = 0. In other words, when *a*_2_ > 1.0, if *x*_1_ − *x*_2_ > 0, then the asymmetric capture rate function is convex, that is, there is a decelerating benefit for the prey species but an accelerating benefit for the predator species. If *x*_1_ − *x*_2_ < 0, then the asymmetric capture rate function is concave, that is, there is an accelerating benefit for the prey species but a decelerating benefit for the predator species (see Fig 1a). Moreover, the trade-off function of prey species *r*(*x*_1_) (the growth rate of prey) is given by

$$\begin{array}{c} r\left(x_{1}\right)=r_{0}+r_{1}(1-x_{1}^{r_{2}}), \end{array}$$ (33)

which is a monotonically decreasing function with respect to *x*_1_. In the following study, we assume that the trade-off curve of prey species is globally concave and fix *r*_0_ = 0.5, *r*_1_ = 1.5, *r*_2_ = 1.5, that is to say, we assume that there is always a weakly accelerating cost in the prey species in the following analysis (see Fig 1b). The trade-off function of predator species *m*(*x*_2_) (the death rate of predators) is given by

$$\begin{array}{c} m\left(x_{2}\right)=m_{0}+m_{1}x_{2}^{m_{2}}, \end{array}$$ (34)

which is a monotonically increasing function with respect to *x*_2_ and *m*_2_ describes the shape and intensity of this trade-off relationship. In particular, when *m*_2_ = 0.9, the trade-off curve of predators is globally weakly concave, that is, there is a weakly decelerating cost in the predator species (see Fig 1c). In contrast, when *m*_2_ > 1.0, the trade-off curve of predators becomes globally convex, that is, there is an accelerating cost in the predator species.

**Fig 2:**
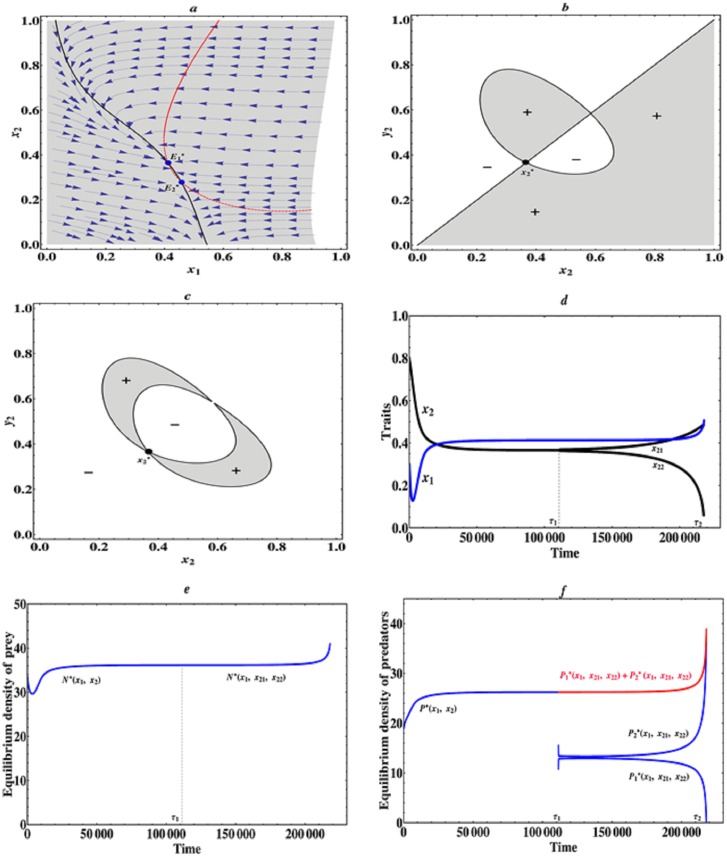
Evolutionary branching and evolutionary murder of predator species. (a) Traits coevolution plot when *a*_2_ = 2.21. The vector fields obtained from deterministic Model (9) indicate directions of coevolution of traits *x*_1_ and *x*_2_. The black curve and red curve indicate respectively isoclines of traits *x*_1_ and *x*_2_. The solid curves indicate evolutionarily singular strategies which are evolutionarily stable, while the dashed curve indicates evolutionarily singular strategy which is not evolutionarily stable. The grey region is feasible phenotype space, in which predator-prey coevolution can occur. (b) Pairwise invasibility plot (PIP) for fixed prey strategy $x_{1}=x_{1}^{*}=0.412$. (c) Mutual invasibility plot (MIP) for fixed prey strategy $x_{1}=x_{1}^{*}=0.412$. (d) Simulated evolutionary tree obtained through simulation of Models (9) and (29) with initial condition (*x*_1_, *x*_2_) = (0.3, 0.8) and *a*_2_ = 2.21. (e) Equilibrium population density of prey species along the coevolutionary trajectory. (f) Equilibrium population densities of predator species along the coevolutionary trajectory. At evolutionary time *τ*_2_, the equilibrium population density of predator 1 becomes zero. The red curve indicates total equilibrium population density of the two predator species. Parameter values: *k* = 0.01, *b* = 0.25, *c* = 0.001, *μ*_1_ = *μ*_2_ = *μ*_21_ = *μ*_22_ = 0.03, *σ*_1_ = *σ*_2_ = *σ*_21_ = *σ*_22_ = 0.03. The other parameter values are the same as described in Fig 1.

In order to explore the impact of intensity of asymmetric interactions on the coevolutionary dynamics of a predator-prey community, when the trade-off curve of predators is globally weakly concave, that is, there is a weakly decelerating cost in the predator species, we take *a*_2_ as the bifurcation parameter in the following numerical analysis and fix other parameter values: *k* = 0.01, *b* = 0.25, *c* = 0.001, *a*_0_ = 0.1, *a*_1_ = 1.0, *r*_0_ = 0.5, *r*_1_ = 1.5, *r*_2_ = 1.5, *m*_0_ = 0.2, *m*_1_ = 0.5, *m*_2_ = 0.9, *μ*_1_ = *μ*_2_ = *μ*_11_ = *μ*_12_ = *μ*_21_ = *μ*_22_ = 0.03, *σ*_1_ = *σ*_2_ = *σ*_11_ = *σ*_12_ = *σ*_21_ = *σ*_22_ = 0.03. Through numerical analysis and computer simulation, we find that when 2.2 ≤ *a*_2_ < 2.3, that is, the asymmetric interactions among prey and predator individuals are relatively weak, then there exists an evolutionarily singular coalition $E_{1}^{*}=\left(x_{1}^{*},x_{2}^{*}\right)$, which is convergence stable, but the predator’s singular strategy $x_{2}^{*}$ is not evolutionarily stable and allows for mutual invasibility nearby, therefore evolutionary branching in the predator species can occur. Particularly, when *a*_2_ = 2.21, we can see that the evolutionarily singular coalition $E_{1}^{*}=\left(0.412,0.365\right)$. In this case, the prey and predators will firstly evolve towards this singular coalition (0.412, 0.365), near this singular coalition the asymmetric predator-prey interactions are relatively weak and there is an accelerating benefit for the predator species, hence the predator species will split up into two different phenotypes and diverge in their traits (see Fig 2a–2d). After branching has occurred in the predator species, we explore the further coevolution of a two-predator-one-prey system. We find that at time *τ*_2_ the equilibrium population density of predator 1 becomes zero, this implies that the upper branch *x*_21_ with a larger trait value becomes extinct (see Fig 2d and 2f), whereas the equilibrium population density of predator 2 with a smaller trait *x*_22_ amounts to a maximum (see Fig 2d and 2f), even though the capture rate of predator 2 is relatively smaller. This may be due to the higher cost of a large trait value, it is difficult for a predator species with a larger phenotypic trait to survive in a certain predator-prey community. In this case, the equilibrium population density of prey species also amounts to its maximum (see Fig 2e). This is an appropriate model for traits evolution, such as arms level, and thus can be seen as an example of evolutionary murder [34, 40]. Therefore, we can see that if there is a weakly decelerating cost and a weakly accelerating benefit in the predator species, then evolutionary branching in the predator species is possible, but due to the higher cost of a large trait value the dimorphic predator species can not coexist on a much longer evolutionary timescale. Finally, the prey and the remaining branch predator 2 with trait values (0.509, 0.054) will evolve to the boundary of feasible phenotype space, because at time *τ*_2_ they lie in the attraction region of another evolutionary equilibrium $E_{2}^{*}=\left(0.456,0.281\right)$ (see Fig 2a and 2d).

**Fig 3:**
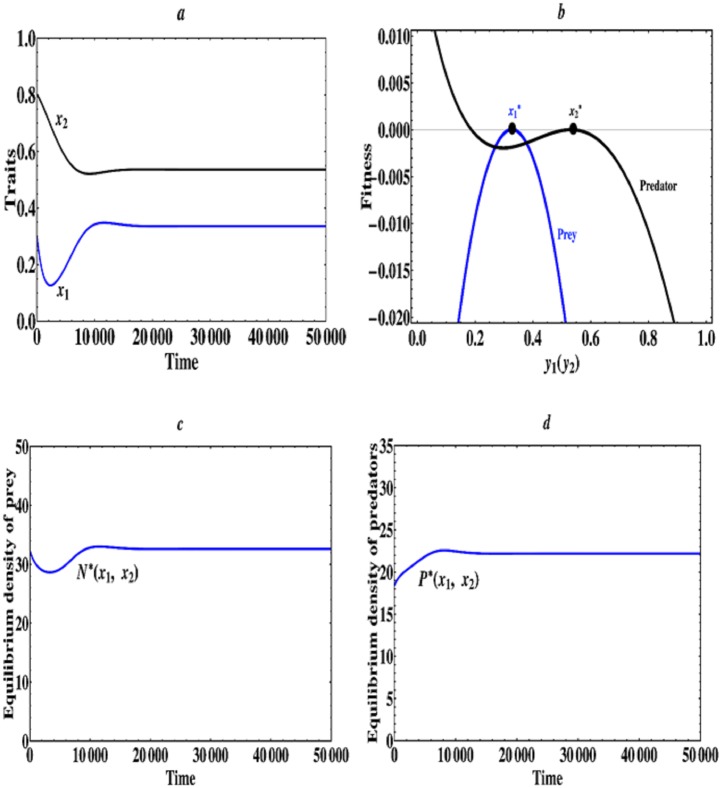
An example of continuously stable strategy. (a) Time series plots obtained through simulation of Model (9) with initial condition (*x*_1_, *x*_2_) = (0.3, 0.8) and *a*_2_ = 2.5. (b) Fitness landscape plots when $\left(x_{1}^{*},x_{2}^{*}\right)=\left(0.335,0.541\right)$ and *a*_2_ = 2.5. (c) Equilibrium population density of prey species as the traits (*x*_1_, *x*_2_) evolve. (d) Equilibrium population density of predator species as the traits (*x*_1_, *x*_2_) evolve. The other parameter values are the same as described in Figs 1 and 2.

When 2.3 ≤ *a*_2_ < 3.35, i.e., the asymmetric predator-prey interactions become a little strong, then there exists an evolutionarily singular coalition $E_{1}^{*}=\left(x_{1}^{*},x_{2}^{*}\right)$, which is both convergence stable and evolutionarily stable, therefore the evolutionarily singular coalition $E_{1}^{*}$ represents an eventual outcome of the coevolutionary process, the prey and predators will evolve towards the singular coalition $E_{1}^{*}$ and come to a halt. Particularly, when *a*_2_ = 2.5, we find that the singular coalition $E_{1}^{*}=\left(0.335,0.541\right)$, in this case, the finally evolutionary outcome contains a monomorphic prey and a monomorphic predator species, which can stably coexist on a much longer evolutionary timescale (see Fig 3a and 3b). The equilibrium population densities of prey and predators are depicted in Fig 3c and 3d when their traits evolve. It can be seen that the population densities of prey and predators finally also reach to a steady state.

**Fig 4:**
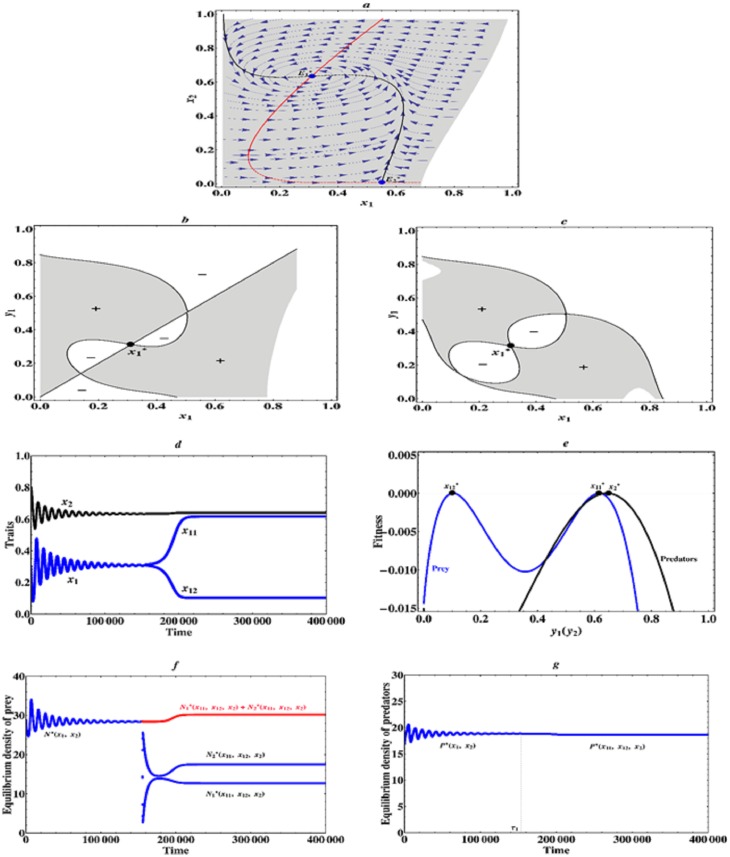
Evolutionary branching and evolutionarily stable coexistence of prey species. (a) Traits coevolution plot when *a*_2_ = 3.8. The vector fields obtained from deterministic Model (9) indicate directions of coevolution of traits *x*_1_ and *x*_2_. The black curve and red curve indicate respectively isoclines of traits *x*_1_ and *x*_2_. The solid curves indicate evolutionarily singular strategies which are evolutionarily stable, while the dashed curves indicate evolutionarily singular strategies which are not evolutionarily stable. The grey region is feasible phenotype space, in which predator-prey coevolution can occur. (b) Pairwise invasibility plot (PIP) for fixed predator strategy $x_{2}=x_{2}^{*}=0.634$. (c) Mutual invasibility plot (MIP) for fixed predator strategy $x_{2}=x_{2}^{*}=0.634$. (d) Simulated evolutionary tree obtained through simulation of Models (9) and (23) with initial condition (*x*_1_, *x*_2_) = (0.3, 0.8) and *a*_2_ = 3.8. (e) Fitness landscape plots when $\left(x_{11}^{*},x_{12}^{*},x_{2}^{*}\right)=\left(0.615,0.105,0.639\right)$ and *a*_2_ = 3.8. (f) Equilibrium population densities of prey species along the coevolutionary trajectory. The red curve indicates total equilibrium population density of the two prey species. (g) Equilibrium population density of predator species along the coevolutionary trajectory. Parameter values: *μ*_1_ = *μ*_2_ = *μ*_11_ = *μ*_12_ = 0.03, *σ*_1_ = *σ*_2_ = *σ*_11_ = *σ*_12_ = 0.03. The other parameter values are the same as described in Figs 1 and 2.

When 3.35 ≤ *a*_2_ < 4.0, that is, the asymmetric interactions among prey and predator individuals become more strong, then there exists an evolutionarily singular coalition $E_{1}^{*}=\left(x_{1}^{*},x_{2}^{*}\right)$, which is convergence stable, but the singular strategy of prey species $x_{1}^{*}$ is not evolutionarily stable and allows for mutual invasibility nearby, therefore evolutionary branching in the prey species can occur. Specifically, when *a*_2_ = 3.8, we find that the evolutionarily singular coalition $E_{1}^{*}=\left(0.309,0.634\right)$, in this case, the prey and predators will firstly evolve towards this singular coalition (0.309, 0.634), near this singular coalition the predator-prey interactions become a little strong, but there is an accelerating benefit for the prey species, so the prey species will branch into two different phenotypes and diverge in their traits (see Fig 4a–4d). After branching has occurred in the prey species, we study the further coevolutionary dynamics of a one-predator-two-prey system. We find that the three species eventually converge to an evolutionarily stable steady state $\left(x_{11}^{*},x_{12}^{*},x_{2}^{*}\right)=\left(0.615,0.105,0.639\right)$, at which they can continue to coexist on a long-term evolutionary timescale (see Fig 4d and 4e). The corresponding equilibrium population densities of prey and predator species are depicted in Fig 4f and 4g. It can be seen that the equilibrium population density of prey 1 with a larger trait *x*_11_ is relatively low, this might be due to the cost of a large trait value. If the prey 1 species have a larger trait *x*_11_, due to the trade-off relationship, they might have a lower growth rate, so their population density is relatively low. However, in this case the predator’s capture rate on them is relatively small and a low population density of prey 1 species can maintain the growth of the whole predator-prey community. Therefore, we can see that if there is a weakly accelerating cost and a relatively strongly accelerating benefit in the prey species, then evolutionary branching in the prey species is possible and the finally evolutionary outcome contains a dimorphic prey and a monomorphic predator species.

**Fig 5:**
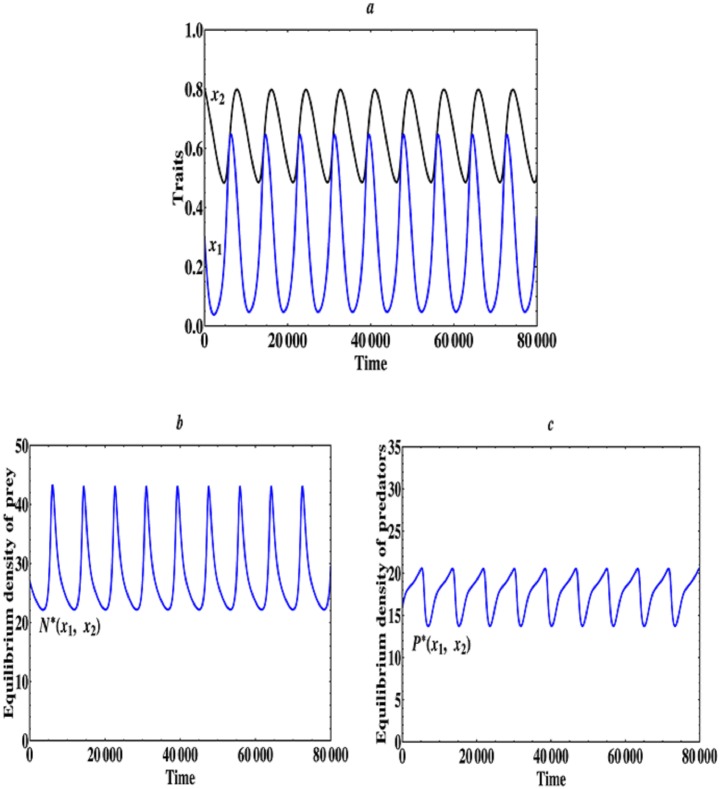
An example of evolutionary cycling. (a) Time series plots obtained through simulation of Model (9) with initial condition (*x*_1_, *x*_2_) = (0.3, 0.8) and *a*_2_ = 5.0. Predators and prey evolve to a stable limit cycle. (b) Equilibrium population density of prey species as the traits (*x*_1_, *x*_2_) evolve. (c) Equilibrium population density of predator species as the traits (*x*_1_, *x*_2_) evolve. The other parameter values are the same as described in Figs 1 and 2.

When 4.0 ≤ *a*_2_ < 11.0, i.e., the asymmetric predator-prey interactions become relatively stronger, then the evolutionarily singular coalition $E_{1}^{*}=\left(x_{1}^{*},x_{2}^{*}\right)$ becomes unstable, in this case we find that the Model (9) admits a Hopf bifurcation, the traits of prey and predators will converge to a stable limit cycle, the limit cycle becomes larger and larger as *a*_2_ increases and ultimately disappears when *a*_2_ approaches to a critical value 11.0. Therefore, we can see that evolutionary cycling is a likely outcome under asymmetric predator-prey interactions. This implies that the relatively stronger asymmetric interactions among prey and predator individuals can keep their phenotypic traits evolving infinitely, which corresponds to a Red Queen dynamics [17]. The specific examples are shown in Fig 5a when *a*_2_ = 5.0. The corresponding equilibrium population densities of prey and predators are depicted in Fig 5b and 5c. It can be seen that the equilibrium population densities of prey and predators also change periodically as their traits (*x*_1_, *x*_2_) evolve.

When *a*_2_ ≥ 11.0, that is, the asymmetric interactions among prey and predator individuals becomes very strong, then the evolutionarily singular coalition $E_{1}^{*}=\left(x_{1}^{*},x_{2}^{*}\right)$ is unstable and there is no limit cycle, the prey and predator species will evolve to the boundary of feasible phenotype space.

**Fig 6:**
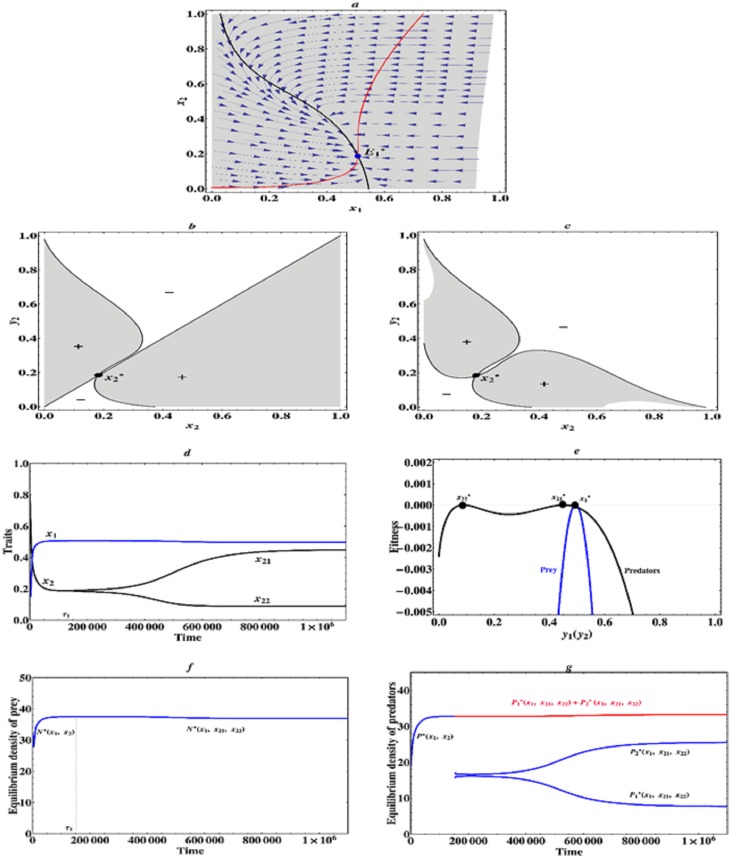
Evolutionarily stable coexistence of a dimorphic predator species. (a) Traits coevolution plot when *a*_2_ = 2.21, *m*_2_ = 1.12. The vector fields obtained from deterministic Model (9) indicate directions of coevolution of traits *x*_1_ and *x*_2_. The black curve and red curve indicate respectively isoclines of traits *x*_1_ and *x*_2_. The solid curves indicate evolutionarily singular strategies which are evolutionarily stable, while the dashed curve indicates evolutionarily singular strategy which is not evolutionarily stable. The grey region is feasible phenotype space, in which predator-prey coevolution can occur. (b) Pairwise invasibility plot (PIP) for fixed prey strategy $x_{1}=x_{1}^{*}=0.507$. (c) Mutual invasibility plot (MIP) for fixed prey strategy $x_{1}=x_{1}^{*}=0.507$. (d) Simulated evolutionary tree obtained through simulation of Models (9) and (29) with initial condition (*x*_1_, *x*_2_) = (0.3, 0.8) and *a*_2_ = 2.21, *m*_2_ = 1.12. (e) Fitness landscape plots when $\left(x_{1}^{*},x_{21}^{*},x_{22}^{*}\right)=\left(0.498,0.45,0.093\right)$ and *a*_2_ = 2.21, *m*_2_ = 1.12. (f) Equilibrium population densities of prey species along the coevolutionary trajectory. The red curve indicates total equilibrium population density of the two prey species. (g) Equilibrium population density of predator species along the coevolutionary trajectory. Parameter values: *μ*_1_ = *μ*_2_ = *μ*_21_ = *μ*_22_ = 0.03, *σ*_1_ = *σ*_2_ = *σ*_21_ = *σ*_22_ = 0.03. The other parameter values are the same as described in Figs 1 and 2.

However, if *a*_2_ = 2.21, but *m*_2_ = 1.12, that is to say, the asymmetric interactions among prey and predator individuals are relatively weak, but the trade-off curve of predators becomes globally weakly convex, in other words, there is a weakly accelerating cost in the predator species, but the cost of a larger trait value becomes relatively low, then there exists an evolutionarily singular coalition $E_{1}^{*}=\left(0.507,0.186\right)$, which is convergence stable, but the singular strategy of predator species $x_{2}^{*}=0.186$ is not evolutionarily stable and allows for mutual invasibility nearby, therefore evolutionary branching in the predator species occurs (see Fig 6a–6d). In this case, the prey and predators will firstly evolve towards this singular coalition (0.507, 0.186), near this singular coalition there is a weakly accelerating benefit for the predator species, hence the predator species branches into two different phenotypes and diverge in their traits. After branching has occurred in the predator species, we further study the coevolutionary dynamics of a two-predator-one-prey system. We find that in this case the three species eventually converge to an evolutionarily stable equilibrium $\left(x_{1}^{*},x_{21}^{*},x_{22}^{*}\right)=\left(0.498,0.45,0.093\right)$, at which they can stably coexist on a much longer timescale of evolution, because this singular strategy is both convergence stable and evolutionarily stable (see Fig 6d and 6e). The corresponding equilibrium population densities of prey and predator species are depicted in Fig 6f and 6g. After branching, it can be seen that due to the cost of a large trait value the equilibrium population density of predators with a larger trait *x*_21_ is relatively low, but finally both the equilibrium population density of prey and total equilibrium population density of the dimorphic predator species amount to their maximum, which is a reasonable model for traits evolution, such as arms level. Therefore, we can see that if there is a weakly accelerating cost and a weakly accelerating benefit in the predator species, then evolutionary branching in the predator species is possible and the finally evolutionary outcome contains a dimorphic predator and a monomorphic prey species.

## Discussion

A central problem in evolutionary biology remains to identify ecological and evolutionary factors that cause species diversity. In this paper, based on a classic predator-prey model, we investigate the impact of asymmetric interactions on coevolutionary dynamics of a predator-prey community. Particularly, we assume that both prey and predators are density-dependent (otherwise, a dimorphic prey (predator) species cannot coexist with a monomorphic predator (prey) species). If the attack ability of predators and the defense ability of prey all can adaptively evolve, but there are certain costs in terms of the predator’s death rate and prey’s growth rate, firstly we find that evolutionary branching in the prey and predator species is possible. For simplicity, we assume that the prey’s trade-off curve is always globally weakly concave, that is to say, there is always a weakly accelerating cost in the prey species. Our results reveal that whether evolutionary branching in the prey and predator species occurs depends not only on the intensity and shape of asymmetric predator-prey interactions, but also on the shape of predator’s trade-off relationship. What’s more, the equilibrium population densities of prey and predator species at the singular strategy are also crucial in determining whether evolutionary branching occurs. In order to determine whether undergoing evolutionary branching, the prey and predator species must balance their costs and benefits as well as their population densities. On the one hand, it is found that when the asymmetric predator-prey interactions are relatively weak, that is, there is a weakly accelerating benefit and a weakly decelerating cost (or a weakly accelerating cost) in the predator species, then evolutionary branching in the predator species is possible, in this case, splitting up into two predator subpopulations will benefit whole predator-prey community. However, under symmetric predator-prey interactions, previous studies showed that in general the predator species will not firstly undergo an evolutionary branching [1, 7, 10, 17, 20, 21]. On the other hand, we find that if the asymmetric predator-prey interactions become relatively strong, that is, there is a weakly accelerating cost and a relatively strongly accelerating benefit in the prey species, then evolutionary branching in the prey species is possible. Moreover, through numerical simulations, we also find that evolutionary branching in the predator and prey species occurs for a large range of strengths of asymmetric interactions when we fix other parameter values. It should be noted that the intraspecific competitions of prey and predator species are essential in determining their evolutionary branching. If there are no intraspecific competitions in the prey and predator species, evolutionary branching in the prey and predator species is not likely to occur. In addition, it would be interesting to discuss the effect of other ecological parameters and trade-off strengths on evolutionary branching of predator or prey species (e.g., the intensity of intraspecific competition of prey species *k*, the conversion efficiency of predators *b* and the trade-off strength *r*_2_). Particularly, in this paper the predator’s functional response is assumed to be linear. Therefore, it would be also interesting to study whether evolutionary branching in the prey and predator species will occur when their functional response is nonlinear [28, 41–45].

After branching has occurred in the predator species, we continue to study the further coevolution of a two-predator-one-prey system and find that if the trade-off curve of predator species is globally weakly concave, that is to say, there is a weakly decelerating cost in the predator species, but the cost of a larger trait value is relatively high, then the further coevolution does not lead to an evolutionarily stable dimorphism in the predator species. In contrast, the dimorphic predator species evolve to the edge of coexistence area, and then the upper branch with a larger trait value becomes extinct, which might be due to the high cost of a larger trait value, the remaining branch continues to evolve to the boundary of the feasible phenotype space, which is a reasonable model for traits evolution, such as arms level, because it is difficult for a predator species with a larger arms to survive in a certain predator-prey community. This phenomenon corresponds to the evolutionary murder [25, 34, 40], which implies that the dimorphism of predator species may be non viability on the timescale of evolution. This phenomenon of evolutionary murder was not observed in the study of coevolution under symmetric predator-prey interaction and other previous works [1, 7, 10, 12, 17, 20, 21, 24]. However, if the trade-off curve of predator species is globally weakly convex, that is to say, there is a weakly accelerating cost in the predator species, but the cost of a larger trait value becomes relatively low, we find that after branching the dimorphic predator species can evolutionarily stably coexist with the monomorphic prey species. Numerical analysis furthermore shows that the dimorphic coevolution maximize the total equilibrium population density of the dimorphic predator species. Therefore, we can see that the property of the predator’s trade-off curve, that is, the cost level of predator species is crucial in determining whether the dimorphic predator species can continue to coexist on the long-term evolutionary timescale. In this study, we find that after branching in the predator species both the predator’s singular strategy and prey’s singular strategy are evolutionarily stable, it remains interesting to investigate under what conditions the evolutionary branching in the predator species will promote evolutionary branching in the prey species.

In another aspect, after branching has occurred in the prey species, we also study the further coevolution of a one-predator-two-prey system and find that for a large range of intensity of such an asymmetric interaction, the prey species will eventually evolve into two different types, which can continue to coexist with the monomorphic predator species on the long-term evolutionary timescale. The eventual outcome of such a coevolutionary process contains a dimorphic prey and a monomorphic predator species. Numerical analysis further shows that the equilibrium population density of prey 1 with a larger trait *x*_11_ is relatively low, this might be due to the the cost of a large trait value. If the prey 1 species have a larger trait *x*_11_, due to the trade-off relationship, they might have a lower growth rate, so their population density is relatively low. However, in this case the predator’s capture rate on them is relatively small and a low population density of prey 1 species can maintain the growth of the whole predator-prey community. Therefore, we can say that if the asymmetric interactions become relatively strong, that is, there is a weakly accelerating cost and a relatively strongly accelerating benefit in the prey species, then the monomorphic prey species can become dimorphic and the dimorphic prey species can evolutionarily stably coexist with a monomorphic predator species. However, such coexistence is not necessarily stable against other life-history traits evolving [37]. Therefore, further research is required in order to identify how robust the coexistence is. In particular, in this study we assume that the prey’s trade-off curve is always globally weakly concave, whereas the eventually coevolutionary outcomes may also depend on the prey’s trade-off shape. It is also interesting to further study the influence of prey’s trade-off shape on evolutionary behaviors of predator and prey and explore whether evolutionary murder of a dimorphic prey species is possible if we take another type of trade-off curve. In addition, under symmetric predator-prey interactions, we found that the evolutionary branching in the prey species can promote the secondary evolutionary branching in the predator species, thus it becomes interesting to investigate under what conditions this phenomenon will occur under asymmetric predator-prey interactions.

More interestingly, we find that there exists a family of stable limit cycles in Model (9) if the asymmetric predator-prey interactions become more strong. The phenotypic traits of prey and predator species will converge to a stable limit cycle. The limit cycle becomes larger and larger as the asymmetric interactions become strong and finally disappears when the asymmetric interactions are strong enough. This implies that predator-prey coevolution can lead to cycles in both traits and equilibrium population densities, even though the prey has a “unidirectional” axis of vulnerability. A “bidirectional” axis of prey vulnerability is not an essential condition that allows for an evolutionary cycling. This phenomenon corresponds to the Red Queen dynamics [17], which can also occur in the coevolution under symmetric predator-prey interactions. To sum up, once a specific asymmetric function *a*(*x*_1_ − *x*_2_) and the life-history trade-off functions *r*(*x*_1_) and *m*(*x*_2_) are given, by using the above method described as in this study, we can easily find that which evolutionary outcome will occur. However, in this study we have not yet found the evolutionary suicide phenomenon although the interactions between prey and predators are asymmetric [40]. Therefore, it is interesting to investigate the evolutionary mechanism of this phenomenon and explore its implication. It should be noted that a dimorphic prey or predator population with asymmetric interactions may have very rich coevolutionary dynamics, a full exploration of which is beyond the scope of this paper.

## Conclusions

In conclusion, this study reveals that the asymmetric interactions promote evolutionary diversity of prey and predator species, which might be an important driving force for speciation. In particular, the strength and shape of asymmetric predator-prey interactions and predator’s trade-off shape are crucial in determining the eventually coevolutionary outcomes. Moreover, the intraspecific competitions of prey and predator species and their equilibrium population densities might also play an important role in determining whether evolutionary branching in the prey or predator species occurs. More interestingly, we find that evolutionary cycling and evolutionarily stable coexistence of a dimorphic prey or predator species are possible outcomes even though the prey has a “unidirectional” axis of vulnerability. However, evolutionary murder of a dimorphic predator species is inevitable if there is a decelerating cost in terms of their mortality rate.

## Supporting Information

S1 Appendix Derivation of invasion fitness *f*_1_(*y*_1_, *x*_1_, *x*_2_). (PDF)

S2 Appendix Invasion implies trait substitution. (PDF)

S3 AppendixGlobal asymptotical stability of $\left(N_{1}^{*}\left(x\right),N_{2}^{*}\left(x\right),P^{*}\left(x\right)\right)$.(PDF)

S4 AppendixGlobal asymptotical stability of $\left(N^{*}\left(x\right),P_{1}^{*}\left(x\right),P_{2}^{*}\left(x\right)\right)$.(PDF)
