# Supplementary material for: Evolutionary Diversification of Prey and Predator Species Facilitated by Asymmetric Interactions
Source: PLoS One. 2016 Sep 29;11(9):e0163753. doi: 10.1371/journal.pone.0163753 (PMC5042472; doi:10.1371/journal.pone.0163753)
Supplement: S1 Appendix — (PDF) [file pone.0163753.s001.pdf]

Evolutionary diversification of prey and predator species facilitated by asymmetric interactions

Jian Zu[1,2,*], Jinliang Wang[3,*], Gang Huang[4]

1 School of Mathematics and Statistics, Xi'an Jiaotong University, Xi'an, 710049, P.R. China
2 Department of Ecology and Evolution, The University of Chicago, Chicago, IL 60637, USA
3 School of Mathematical Science, Heilongjiang University, Harbin, 150080, P.R. China
4 School of Mathematics and Physics, China University of Geosciences, Wuhan, 430074, P.R. China

* Corresponding author: jianzu@xjtu.edu.cn; jinliangwang@hlju.edu.cn

S1 Appendix. Derivation of invasion fitness $f_1(y_1, x_1, x_2)$.

In this appendix, we explain how to derive the invasion fitness $f_1(y_1, x_1, x_2)$ for a mutant prey species. When a mutant prey with a different trait y_1 appears in the resident predator-prey community, the resident-mutant population dynamics is given by

$$\begin{cases} \dfrac{dP}{dt} = ba(x_1 - x_2)NP + ba(y_1 - x_2)N_m P - m(x_2)P - cP^2, \\ \dfrac{dN}{dt} = r(x_1)N - k(N + N_m)N - a(x_1 - x_2)NP, \\ \dfrac{dN_m}{dt} = r(y_1)N_m - k(N + N_m)N_m - a(y_1 - x_2)N_m P, \end{cases} \tag{1}$$

where N_m is the population density of mutant prey at time t. One equilibrium of model (1) is $(P^*(x_1, x_2), N^*(x_1, x_2), 0)$, where $P^*(x_1, x_2)$ and $N^*(x_1, x_2)$ are described in (4) of main text. It is assumed that mutations occur infrequently, thus just after the small and rare mutations, the resident and mutant prey and predators are close to the ecological equilibrium $(P^*(x_1, x_2), N^*(x_1, x_2), 0)$. If this equilibrium is unstable, then the population density of mutant prey will initially increase, that is to say, the mutant prey can invade. Therefore, we perform a stability analysis on this equilibrium.

The Jacobian matrix J_2 of model (1) evaluated at this equilibrium $(P^*(x_1, x_2), N^*(x_1, x_2), 0)$ is given by

$$J_2 = \begin{bmatrix} \mathbf{J_{res}} & \mathbf{J_3} \\ \mathbf{0} & \mathbf{J_{mut}} \end{bmatrix},$$

where

$$\mathbf{J_{res}} = \begin{bmatrix} -cP^*(x_1, x_2) & ba(x_1 - x_2)P^*(x_1, x_2) \\ -a(x_1 - x_2)N^*(x_1, x_2) & -kN^*(x_1, x_2) \end{bmatrix}, \ \mathbf{J_3} = \begin{bmatrix} ba(y_1 - x_2)P^*(x_1, x_2) \\ -kN^*(x_1, x_2) \end{bmatrix},$$

$\mathbf{0} = (0, 0)$ and $\mathbf{J_{mut}} = (r(y_1) - kN^*(x_1, x_2) - a(y_1 - x_2)P^*(x_1, x_2))$. Because J_2 is a block triangular, and the ecological equilibrium $(P^*(x_1, x_2), N^*(x_1, x_2))$ is globally

asymptotically stable, that is, the two eigenvalues of $\mathbf{J_{res}}$ have negative real parts, the stability of equilibrium $(P^*(x_1, x_2), N^*(x_1, x_2), 0)$ is determined by the single element of $\mathbf{J_{mut}}$, which we define as $f_1(y_1, x_1, x_2)$, i.e.,

$$f_1(y_1, x_1, x_2) = r(y_1) - kN^*(x_1, x_2) - a(y_1 - x_2)P^*(x_1, x_2). \tag{2}$$

We can see that if $f_1(y_1, x_1, x_2) > 0$, then the ecological equilibrium $(P^*(x_1, x_2), N^*(x_1, x_2), 0)$ is unstable, the population density of mutant prey will initially increase, i.e., the mutant prey can invade. Therefore, $f_1(y_1, x_1, x_2)$ is defined as the invasion fitness for a mutant prey species.
