# Supplementary material for: Evolutionary Diversification of Prey and Predator Species Facilitated by Asymmetric Interactions
Source: PLoS One. 2016 Sep 29;11(9):e0163753. doi: 10.1371/journal.pone.0163753 (PMC5042472; doi:10.1371/journal.pone.0163753)
Supplement: S2 Appendix — (PDF) [file pone.0163753.s002.pdf]

# Evolutionary diversification of prey and predator species facilitated by asymmetric interactions

Jian Zu[1,2,*], Jinliang Wang[3,*], Gang Huang[4]

**1** School of Mathematics and Statistics, Xi'an Jiaotong University, Xi'an, 710049, P.R. China
**2** Department of Ecology and Evolution, The University of Chicago, Chicago, IL 60637, USA
**3** School of Mathematical Science, Heilongjiang University, Harbin, 150080, P.R. China
**4** School of Mathematics and Physics, China University of Geosciences, Wuhan, 430074, P.R. China

* Corresponding author: jianzu@xjtu.edu.cn; jinliangwang@hlju.edu.cn

## S2 Appendix. Invasion implies trait substitution.

In this appendix, by using the method of Lyapunov function, we show that a successful invasion generally cause a trait substitution. First, by simply exchanging the roles of the resident and mutant prey, we obtain another invasion fitness $\widetilde{f}_1(x_1, y_1, x_2)$, i.e.,

$$\widetilde{f}_1(x_1, y_1, x_2) = r(x_1) - kN_m^*(y_1, x_2) - a(x_1 - x_2)P^*(y_1, x_2), \quad (1)$$

where $N_m^*(y_1, x_2)$ and $P^*(y_1, x_2)$ are described as in (4) of main text by simply replacing $x_1$ with $y_1$. Because the traits $y_1$ and $x_1$ are very similar to each other, expanding $f_1(y_1, x_1, x_2)$ in Taylor series around $y_1 = x_1$ and using the fact that $f_1(x_1, x_1, x_2) = 0$, we get

$$\begin{aligned} f_1(y_1, x_1, x_2) &= f_1(x_1, x_1, x_2) + \left.\frac{\partial f_1(y_1, x_1, x_2)}{\partial y_1}\right|_{y_1 = x_1} (y_1 - x_1) + O\left(|y_1 - x_1|^2\right) \\ &= (r'(x_1) - a'(x_1 - x_2)P^*(x_1, x_2))(y_1 - x_1) + O\left(|y_1 - x_1|^2\right). \end{aligned} \quad (2)$$

Similarly, expanding $\widetilde{f}_1(x_1, y_1, x_2)$ in Taylor series around $y_1 = x_1$ and using the fact that $\widetilde{f}_1(x_1, x_1, x_2) = 0$, we obtain

$$\begin{aligned} \widetilde{f}_1(x_1, y_1, x_2) &= \widetilde{f}_1(x_1, x_1, x_2) + \left.\frac{\partial \widetilde{f}_1(x_1, y_1, x_2)}{\partial y_1}\right|_{y_1 = x_1} (y_1 - x_1) + O\left(|y_1 - x_1|^2\right) \\ &= -(r'(x_1) - a'(x_1 - x_2)P^*(x_1, x_2))(y_1 - x_1) + O\left(|y_1 - x_1|^2\right). \end{aligned} \quad (3)$$

Thus, from (2) and (3), it can be seen that generally for $y_1$ adequately close to $x_1$ and $x_1$ is not an evolutionarily singular strategy, then $f_1(y_1, x_1, x_2)$ and $\widetilde{f}_1(x_1, y_1, x_2)$ are of opposite sign.

Next, by using the method of Lyapunov function, we show that if $x_1$ is not an evolutionarily singular strategy and $f_1(y_1, x_1, x_2) > 0$, then the boundary equilibrium $(P^*(y_1, x_2), 0, N_m^*(y_1, x_2))$ of the model (1) in S1 Appendix is globally asymptotically stable in $\mathbf{R}_\dagger^3 = \{P > 0, N \geq 0, N_m > 0\}$, which implies that a successful invasion cause

a trait substitution. For simplicity, we use $P^*$ and $N_m^*$ instead of $P^*(y_1, x_2)$ and $N_m^*(y_1, x_2)$. The Lyapunov function is as following

$$V_1 = \left(P - P^* - P^* \ln \frac{P}{P^*}\right) + bN + b\left(N_m - N_m^* - N_m^* \ln \frac{N_m}{N_m^*}\right). \tag{4}$$

It is clear that $V_1 \geq 0$ and the equality holds only for $(P, N, N_m) = (P^*, 0, N_m^*)$. Furthermore, the time derivative of $V_1$ along solutions of model (1) in S1 Appendix is give by

$$\begin{aligned}
\frac{dV_1}{dt} &= (P - P^*)\frac{1}{P}\frac{dP}{dt} + b\frac{dN}{dt} + b\left(N_m - N_m^*\right)\frac{1}{N_m}\frac{dN_m}{dt} \\
&= (P - P^*)\left(ba(x_1 - x_2)N + ba(y_1 - x_2)N_m - m(x_2) - cP\right) \\
&\quad + bN\left(r(x_1) - k(N + N_m) - a(x_1 - x_2)P\right) \\
&\quad + b\left(N_m - N_m^*\right)\left(r(y_1) - k(N + N_m) - a(y_1 - x_2)P\right) \\
&= (P - P^*)\left(ba(x_1 - x_2)N + ba(y_1 - x_2)(N_m - N_m^*) - c(P - P^*)\right) \\
&\quad + bN\left(r(x_1) - kN_m^* - a(x_1 - x_2)P^*\right) \\
&\quad + bN\left(-kN - k(N_m - N_m^*) - a(x_1 - x_2)(P - P^*)\right) \\
&\quad + b\left(N_m - N_m^*\right)\left(-kN - k(N_m - N_m^*) - a(y_1 - x_2)(P - P^*)\right) \\
&= bN\widetilde{f}_1(x_1, y_1, x_2) - c(P - P^*)^2 - bk(N + N_m - N_m^*)^2.
\end{aligned} \tag{5}$$

From the proof of the first part, we can see that if $f_1(y_1, x_1, x_2) > 0$, then $\widetilde{f}_1(x_1, y_1, x_2) < 0$. Thus, if $f_1(y_1, x_1, x_2) > 0$, we have $dV_1/dt \leq 0$ in $\mathbf{R}_+^3$. Moreover, it can be seen that $dV_1/dt = 0$ if and only if $(P, N, N_m) = (P^*, 0, N_m^*)$. By the invariance principle of Lyapunov-LaSalle, we can see that if $x_1$ is not an evolutionarily singular strategy and $f_1(y_1, x_1, x_2) > 0$, then the boundary equilibrium $(P^*(y_1, x_2), 0, N_m^*(y_1, x_2))$ is globally asymptotically stable.

Similarly, it can be shown that if $f_2(y_2, x_1, x_2) > 0$ and the trait $x_2$ is not an evolutionarily singular strategy, then a successful invasion will cause a trait substitution of the predator species.
