# Supplementary material for: Evolutionary Diversification of Prey and Predator Species Facilitated by Asymmetric Interactions
Source: PLoS One. 2016 Sep 29;11(9):e0163753. doi: 10.1371/journal.pone.0163753 (PMC5042472; doi:10.1371/journal.pone.0163753)
Supplement: S3 Appendix — (PDF) [file pone.0163753.s003.pdf]

Evolutionary diversification of prey and predator species facilitated by asymmetric interactions

Jian Zu[1,2,*], Jinliang Wang[3,*], Gang Huang[4]

1 School of Mathematics and Statistics, Xi'an Jiaotong University, Xi'an, 710049, P.R. China
2 Department of Ecology and Evolution, The University of Chicago, Chicago, IL 60637, USA
3 School of Mathematical Science, Heilongjiang University, Harbin, 150080, P.R. China
4 School of Mathematics and Physics, China University of Geosciences, Wuhan, 430074, P.R. China

* Corresponding author: jianzu@xjtu.edu.cn; jinliangwang@hlju.edu.cn

S3 Appendix. Global asymptotical stability of $(N_1^*(\mathbf{x}), N_2^*(\mathbf{x}), P^*(\mathbf{x}))$.

In this appendix, we use the Lyapunov function method to show that if b_i $(i = 1, 2, 3)$ in (18) of main text are positive, then the ecological equilibrium $(N_1^*(\mathbf{x}), N_2^*(\mathbf{x}), P^*(\mathbf{x}))$ of model (17) of main text is globally asymptotically stable in $\mathbf{R}_\dagger^3 = \{N_1 > 0, N_2 > 0, P > 0\}$. For simplicity of notation, (N_1^*, N_2^*, P^*) is used to instead of $(N_1^*(\mathbf{x}), N_2^*(\mathbf{x}), P^*(\mathbf{x}))$. The Lyapunov function is as following

$$V_2 = b\left(N_1 - N_1^* - N_1^* \ln \frac{N_1}{N_1^*}\right) + b\left(N_2 - N_2^* - N_2^* \ln \frac{N_2}{N_2^*}\right) + \left(P - P^* - P^* \ln \frac{P}{P^*}\right). \tag{1}$$

We can see that $V_2 \geq 0$ and the equality holds only if $(N_1, N_2, P) = (N_1^*, N_2^*, P^*)$. The time derivative of V_2 along solutions of model (17) of main text is given by

$$\begin{aligned}
\frac{dV_2}{dt} &= b\left(N_1 - N_1^*\right)\frac{1}{N_1}\frac{dN_1}{dt} + b\left(N_2 - N_2^*\right)\frac{1}{N_2}\frac{dN_2}{dt} + \left(P - P^*\right)\frac{1}{P}\frac{dP}{dt} \\
&= b\left(N_1 - N_1^*\right)\left(r(x_{11}) - k(N_1 + N_2) - a(x_{11} - x_2)P\right) \\
&\quad + b\left(N_2 - N_2^*\right)\left(r(x_{12}) - k(N_1 + N_2) - a(x_{12} - x_2)P\right) \\
&\quad + \left(P - P^*\right)\left(ba(x_{11} - x_2)N_1 + ba(x_{12} - x_2)N_2 - m(x_2) - cP\right) \\
&= b\left(N_1 - N_1^*\right)\left(-k(N_1 - N_1^*) - k(N_2 - N_2^*) - a(x_{11} - x_2)(P - P^*)\right) \\
&\quad + b\left(N_2 - N_2^*\right)\left(-k(N_1 - N_1^*) - k(N_2 - N_2^*) - a(x_{12} - x_2)(P - P^*)\right) \\
&\quad + \left(P - P^*\right)\left(ba(x_{11} - x_2)(N_1 - N_1^*) + ba(x_{12} - x_2)(N_2 - N_2^*) - c(P - P^*)\right) \\
&= -bk\left((N_1 - N_1^*) + (N_2 - N_2^*)\right)^2 - c(P - P^*)^2.
\end{aligned} \tag{2}$$

From (2), it can be seen that if there is a positive ecological equilibrium $(N_1^*(\mathbf{x}), N_2^*(\mathbf{x}), P^*(\mathbf{x}))$, then $dV_2/dt \leq 0$ in $\mathbf{R}_\dagger^3$. Moreover, $dV_2/dt = 0$ if and only if $(N_1, N_2, P) = (N_1^*, N_2^*, P^*)$. Thus, by the invariance principle of Lyapunov-LaSalle, we can see that if b_i $(i = 1, 2, 3)$ in (18) of main text are positive, then the ecological equilibrium $(N_1^*(\mathbf{x}), N_2^*(\mathbf{x}), P^*(\mathbf{x}))$ is globally asymptotically stable.
