# Supplementary material for: Evolutionary Diversification of Prey and Predator Species Facilitated by Asymmetric Interactions
Source: PLoS One. 2016 Sep 29;11(9):e0163753. doi: 10.1371/journal.pone.0163753 (PMC5042472; doi:10.1371/journal.pone.0163753)
Supplement: S4 Appendix — (PDF) [file pone.0163753.s004.pdf]

Evolutionary diversification of prey and predator species facilitated by asymmetric interactions

Jian Zu[1,2,*], Jinliang Wang[3,*], Gang Huang[4]

1 School of Mathematics and Statistics, Xi'an Jiaotong University, Xi'an, 710049, P.R. China
2 Department of Ecology and Evolution, The University of Chicago, Chicago, IL 60637, USA
3 School of Mathematical Science, Heilongjiang University, Harbin, 150080, P.R. China
4 School of Mathematics and Physics, China University of Geosciences, Wuhan, 430074, P.R. China

* Corresponding author: jianzu@xjtu.edu.cn; jinliangwang@hlju.edu.cn

S4 Appendix. Global asymptotical stability of $(N^*(\mathbf{x}), P_1^*(\mathbf{x}), P_2^*(\mathbf{x}))$.

In this appendix, we use the method of Lyapunov function to prove that if c_i $(i = 1, 2, 3)$ in (26) of main text are positive, then the ecological equilibrium $(N^*(\mathbf{x}), P_1^*(\mathbf{x}), P_2^*(\mathbf{x}))$ of model (25) of main text is globally asymptotically stable in $\mathbf{R}^3_\dagger = \{N > 0, P_1 > 0, P_2 > 0\}$. For simplicity of notation, (N^*, P_1^*, P_2^*) is used to instead of $(N^*(\mathbf{x}), P_1^*(\mathbf{x}), P_2^*(\mathbf{x}))$. The Lyapunov function is given by

$$V_3 = b\left(N - N^* - N^* \ln\frac{N}{N^*}\right) + \left(P_1 - P_1^* - P_1^* \ln\frac{P_1}{P_1^*}\right) + \left(P_2 - P_2^* - P_2^* \ln\frac{P_2}{P_2^*}\right). \quad (1)$$

It is easy to show that $V_3 \geq 0$ and the equality holds only if $(N, P_1, P_2) = (N^*, P_1^*, P_2^*)$. The time derivative of V_3 along the solutions of model (25) of main text is given by

$$\begin{aligned}
\frac{dV_3}{dt} &= b\,(N - N^*)\,\frac{1}{N}\frac{dN}{dt} + (P_1 - P_1^*)\,\frac{1}{P_1}\frac{dP_1}{dt} + (P_2 - P_2^*)\,\frac{1}{P_2}\frac{dP_2}{dt} \\
&= b\,(N - N^*)\,(r(x_1) - kN - a(x_1 - x_{21})P_1 - a(x_1 - x_{22})P_2) \\
&\quad + (P_1 - P_1^*)\,(ba(x_1 - x_{21})N - m(x_{21}) - c(P_1 + P_2)) \\
&\quad + (P_2 - P_2^*)\,(ba(x_1 - x_{22})N - m(x_{22}) - c(P_1 + P_2)) \\
&= b\,(N - N^*)\,(-k(N - N^*) - a(x_1 - x_{21})(P_1 - P_1^*) - a(x_1 - x_{22})(P_2 - P_2^*)) \\
&\quad + (P_1 - P_1^*)\,(ba(x_1 - x_{21})(N - N^*) - c(P_1 - P_1^*) - c(P_2 - P_2^*)) \\
&\quad + (P_2 - P_2^*)\,(ba(x_1 - x_{22})(N - N^*) - c(P_1 - P_1^*) - c(P_2 - P_2^*)) \\
&= -bk(N - N^*)^2 - c\,((P_1 - P_1^*) + (P_2 - P_2^*))^2 .
\end{aligned} \quad (2)$$

From (2), we can see that if there is a positive ecological equilibrium $(N^*(\mathbf{x}), P_1^*(\mathbf{x}), P_2^*(\mathbf{x}))$, then $dV_3/dt \leq 0$ in $\mathbf{R}^3_\dagger$. In addition, it can be seen that $dV_3/dt = 0$ if and only if $(N, P_1, P_2) = (N^*, P_1^*, P_2^*)$. Therefore, by the Lyapunov-LaSalle's invariance principle, we can see that if c_i $(i = 1, 2, 3)$ in (26) of main text are positive, then the ecological equilibrium $(N^*(\mathbf{x}), P_1^*(\mathbf{x}), P_2^*(\mathbf{x}))$ is globally asymptotically stable.
